# Chinese herbal injections combined with EGFR-TKIs for intervention of non-small cell lung cancer: a systematic review and meta-analysis

**DOI:** 10.3389/fphar.2025.1670501

**Published:** 2025-11-19

**Authors:** Zichun Yuan, Mei Yan, Tongtong Wu, Ganlin Zhang, Guowang Yang, Xiaomin Wang

**Affiliations:** 1 Graduate School, Beijing University of Chinese Medicine, Beijing, China; 2 Department of Oncology, Beijing Hospital of Traditional Chinese Medicine, Capital Medical University, Beijing, China

**Keywords:** Chinese herbal injections, epidermal growth factor receptor tyrosine kinase inhibitors, non-small cell lung cancer, meta-analysis, systematic review

## Abstract

**Objective:**

To serve as a clinical reference, we conducted a meta-analysis to assess and compare the efficacy and safety of combining Chinese herbal injections (CHIs) with epidermal growth factor receptor tyrosine kinase inhibitors (EGFR-TKIs) for treating advanced EGFR-mutated non-small cell lung cancer (NSCLC).

**Methods:**

Predetermined databases contained randomized controlled studies comparing CHIs + EGFR-TKIs to EGFR-TKIs alone. The Cochrane Reviewer’s Handbook for Systematic Reviews of Interventions was used to evaluate study methodology. To assess the effects of CHIs on patients with NSCLC receiving EGFR-TKIs, R (version 4.4.0) and RevMan (version 5.4) were used to conduct a meta-analysis using the Mantel–Haenszel or Inverse Variance method. The quality of results was evaluated using the Grade of Recommendations Assessment, Development and Evaluation (GRADE) method.

**Results:**

The 13 qualifying trials included 899 subjects. The objective response rate (ORR) was significantly increased by Kanglaite injection (RR = 1.52, 95% confidence interval [CI]: 1.07–2.15, *p* = 0.02). The disease control rate (DCR) was influenced by Kanglaite injection (RR = 1.14, 95% CI: 1.01–1.29, *p* = 0.04) and Aidi injection (RR = 1.23, 95% CI: 1.02–1.48, *p* = 0.03). Shenmai injection + EGFR-TKIs reduced dermatologic toxicities (RR = 0.35, 95% CI: 0.18–0.69, *p* = 0.002). The combination of CHIs with EGFR-TKIs improved the expression of CD3^+^ (standardized mean difference [SMD] = 1.38, 95% CI: 0.46–2.30, *p* = 0.003), CD4^+^ (SMD = 1.08, 95% CI: 0.51–1.65, *p* = 0.0002), and the CD4^+^/CD8^+^ ratio (SMD = 0.96, 95% CI: 0.54–1.38, *p* < 0.00001) compared to EGFR-TKIs alone. The GRADE method revealed that most outcomes exhibited low certainty evidence.

**Conclusion:**

The combination of CHIs and EGFR-TKIs improved ORR, DCR, adverse effects, and immune function in patients with NSCLC. Due to limitations in the assessed research, high-quality clinical studies with rigorous designs are required to confirm the findings.

**Systematic Review:**

https://www.crd.york.ac.uk/PROSPERO/view/CRD42024561845, Identifier CRD42024561845.

## 1 Introduction

Lung cancer is the leading cause of cancer-related deaths worldwide, with non-small cell lung cancer (NSCLC) accounting for approximately 85% of all documented cases (Sung et al., 2021). Comprehensive surgical therapy is used for early-stage NSCLC, whereas intermediate- and late-stage NSCLC are frequently managed with a combination of radiotherapy, chemotherapy, targeted therapy, and immunotherapy. Activating mutations in the epidermal growth factor receptor (EGFR) gene were detected in 15%–20% of patients with NSCLC, primarily in adenocarcinoma patients without smoking history and Asian patients (Jänne et al., 2005). Extensive phase III clinical studies (Tan et al., 2016; Rosell et al., 2012; Sequist et al., 2013; Wu et al., 2015) have validated EGFR tyrosine kinase inhibitors (EGFR-TKIs) as the preferred first-line treatment for EGFR mutant NSCLC, outperforming chemotherapy in terms of progression-free survival (PFS), objective response rate (ORR), and quality of life (QOL). Currently, erlotinib, gefitinib, and icotinib are categorized as first-generation EGFR-TKIs, afatinib and dacomitinib as second-generation, and osimertinib and almonertinib as third-generation EGFR-TKIs (Koulouris et al., 2022). It is important to acknowledge that targeted therapy may induce adverse effects, leading to multi-organ toxicity or malfunction, as well as the unavoidable issue of drug resistance, complicating subsequent treatment options.

Traditional Chinese medicine (TCM) posits that cancer is primarily caused by an imbalance of Yin and Yang, a deficiency of Zhengqi, and an excess of Xieqi. Therefore, the therapeutic principles reside in Fuzheng (harmonization) and Quxie (removal of pathogenic elements), as well as supplementation and promotion of Yin-Yang rebalancing (Li et al., 2025). TCM inhibits tumor cell proliferation, metastasis, angiogenesis, and lymphangiogenesis, and promotes tumor cell death (Wang et al., 2021). Early studies have demonstrated that Chinese herbal injections (CHIs), a preparation of botanical extracts, are widely used in the treatment of cancer. For instance, Xiaoaiping injection alters the cell cycle, mitogen-activated protein kinase signaling pathway, and regulatory proteins to inhibit the proliferation of human esophageal cancer cells. Kangai injection incorporates Astragalus, ginseng, and kurorinone as primary extracts and is extensively used in treating lung, gastric, and liver cancers. CHIs and targeted therapies demonstrate synergistic effects. For example, Complex Ku Shen injection was administered alongside sorafenib, a TKI, to treat hepatocellular carcinoma (Li et al., 2025).

TCM has gained significant popularity as an alternative and supplementary treatment for advanced NSCLC (Jiao et al., 2019), potentially mitigating the adverse effects of EGFR-TKIs (Hu et al., 2021) and improving QOL. Numerous network meta-analyses of CHIs in conjunction with chemotherapy have been performed (Wen et al., 2023; Li et al., 2022). Integrating several types of CHIs with chemotherapy is useful for increasing response rates, improving QOL, and strengthening immune function. Given the increasing use of targeted therapy in lung cancer treatment, we specifically investigated the role of CHIs in integrating targeted therapy. In recent years, advances in clinical research on CHIs in conjunction with EGFR-TKIs have yielded new evidence-based medical findings. A comprehensive assessment of the efficacy and safety of combining CHIs with EGFR-TKIs is necessary to establish a foundation for their clinical use in NSCLC treatment.

## 2 Methods

This network meta-analysis was performed according to the Preferred Reporting Items for Systematic Reviews and Meta-Analyses (PRISMA) (Supplementary Table S1) (Hutton et al., 2015). The protocol is registered in the Prospective Register of Systematic Reviews (PROSPERO) (CRD42024561845). Detailed documents are provided in Supplementary Materials.

### 2.1 Standard evaluation of the composition of CHIs

Phytochemical characterization was conducted according to the ConPhyMP consensus recommendations for medicinal plant extract analysis to maintain methodological rigor. We standardized botanical nomenclature for all medical plant using the Medical Plant Name Service (http://mpns.kew.org/mpns-portal/) and Plants of the World Online (http://www.plantsoftheworldonline.org). Composition summaries were organized according to the Four Pillars of Best Practice in Ethnopharmacology (Table 1). Additional information regarding the CHIs is listed in Supplementary Table S2.

**TABLE 1:** Composition and taxonomical information of botanical drugs used in CHIs.

Pharmacopeial drug name	Composition	Source species (family)	Level of reporting in the original study
Kanglaite injection	Coix lacryma-jobi var.ma-yuen (Rom.Caill.) Stapf (Yiyiren in Chinese)	Poaceae	Inadequate
Aidi injection	Panax ginseng C.A.Mey (Renshen in Chinese) Astragalus mongholicus Bunge (Huangqi in Chinese) Eleutherococcus senticosus (Rupr. and Maxim.) Maxim. (Ciwujia in Chinese)	Araliaceae Fabaceae	Inadequate
Shenmai injection	Panax ginseng C.A.Mey (Hongshen in Chinese) Ophiopogon japonicus (Thunb.) Ker Gawl. (Maidong in Chinese)	Araliaceae Asparagaceae	Inadequate
Shenfu injection	Panax ginseng C.A.Mey (Hongshen in Chinese) Aconitum carmichaelii Debeaux (Heifupian in Chinese)	Araliaceae Ranunculaceae	Inadequate
Xiaoaiping injection	Gongronemopsis tenacissima (Roxb.) S.Reuss, Liede and Meve (Tongguanteng in Chinese)	Apocynaceae	Inadequate
Elemene emulsion injection	Curcuma aromatica Salisb. (Wenyujin in Chinese)	Zingiberaceae	Inadequate

### 2.2 Data sources and searches

We searched Chinese databases, including the China National Knowledge Infrastructure (CNKI), Chinese Biological Medicine Database (CBM), Wanfang Database, and VIP Database for Chinese Technical Periodicals, as well as English databases, including MEDLINE (via PubMed), Embase (via Ovid), and Cochrane Library, to find related articles up to 10 October 2024. Two investigators independently searched for articles using a mix of primary search terms “NSCLC,” “EGFR-TKIs,” and “Chinese herbal injections” within the context of “randomized controlled trial.” The search approach is listed in Supplementary Table S3.

### 2.3 Study selection

Randomized controlled trials that were published and met the following requirements were included: Trials including participants with advanced (stage III/IV) histology or cytological confirmation with EGFR-activating mutations in NSCLC; the baseline circumstances of the experimental and control groups did not differ significantly (*p* > 0.05); the experimental group was administered CHIs + EGFR-TKIs, while the control group received only EGFR-TKIs, including the first-generation (Gefitinib) to third-generation (Osimertinib); both groups received the same supportive care, without other anti-tumor therapies; ORR and disease control rate (DCR) were reported as primary outcomes, while secondary outcomes included QOL, adverse effects, and immune function.

The exclusion criteria were as follows: Studies involving patients with additional primary malignancies; studies in which treatments were coupled with surgery, radiation, chemotherapy, or other TCM therapies; studies in which the name, dosage, and treatment regimen of CHIs and EGFR-TKIs were not specified; duplicate studies, non-randomized controlled trials, or studies using flawed randomization procedures; studies lacking credible effectiveness and safety evidence or a clear delineation of the efficacy evaluation criteria.

### 2.4 Outcome indicators and evaluation criteria

ORR and DCR were assessed according to the World Health Organization evaluation criteria or the response evaluation criteria in solid tumors. The responses were classified as complete relief (CR), partial remission (PR), stable disease (SD), or progressive disease (PD). ORR was calculated as (CR + PR)/total number of cases × 100%, and DCR was calculated as (CR + PR + SD)/total number of cases × 100%.

The European Organization for Research and Treatment of Cancer Quality of Life Group (EORTC QLG Core Questionnaire [EORTC QLQ-C30]) or Karnofsky Performance Scale (KPS) was used to assess the patient’s QOL. EORTC QLQ-C30 can be divided into three aspects: Functional areas, symptom fields, and overall health status. The average score of each aspect was compared between the experimental and control groups to validate the improvement in QOL.

Common adverse reactions associated with targeted therapy were evaluated, including gastrointestinal toxicities (nausea and vomiting), dermatologic toxicities (erythema and pruritus), and hepatic insufficiency.

Furthermore, the efficacy of CHIs + EGFR-TKIs was estimated using the incidence of changes in the cellular immune index (CD3^+^, CD4^+^, CD8^+^, and CD4^+^/CD8^+^ ratio). Immunocytochemistry or flow cytometry was used to analyze immunological changes in the included studies.

### 2.5 Data obtaining and quality evaluation

The general features of qualifying studies were independently retrieved and cross-checked by two researchers. A third researcher was consulted to resolve any disagreements. The first author’s name, year of publication, number of patients in each group, patients’ gender, age, and tumor stage, specifics of the intervention (targeted therapy regimens or treatment course), and the outcome indicators were obtained from each paper. Two reviewers assessed the methodological quality of each included RCT according to the Cochrane Collaboration’s risk of bias criteria (Higgins et al., 2019), based on the following factors: Random sequence generation (selection bias), allocation concealment (selection bias), blinding of participants and personnel (performance bias), blinding of outcome data (detection bias), incomplete outcome data (attrition bias), selective reporting (reporting bias), and other biases.

### 2.6 Statistical analysis

R (version 4.4.0) was used for meta-analysis to evaluate the overall impact of CHIs on NSCLC. The analysis was conducted using the R package “meta.” Standardized mean differences (SMDs) and risk ratios (RRs) were used to investigate the effect size for continuous and dichotomous outcomes, respectively. The effect size and 95% confidence interval (CI) were used to calculate RRs and SMDs. *P* < 0.05 indicated statistical significance. The Mantel–Haenszel method was used for dichotomous outcomes with low event incidence, while the inverse variance method was deemed more appropriate due to its broader applicability and capacity to evaluate heterogeneity. A fixed or random-effects model was selected based on the heterogeneity among trials and evaluated using the I^2^ statistic and conceptual heterogeneity. The extent of heterogeneity among studies within the overall variation was quantitatively delineated using I^2^. I^2^ > 50% indicated a significant level of variability. However, I^2^ exhibits certain limitations. It fails to identify the source of heterogeneity and exhibits minimal test power when the number of included studies is limited. The consistency of the included studies on population, intervention, comparison, and outcome (PICO) is crucial when discussing heterogeneity. If the studies exhibit consistency in PICO, a fixed-effect model is preferred. Conversely, a random-effect model is more suitable when there is an anticipated and justifiable variation in PICO among studies. RevMan (version 5.4) was used to validate the results and generate forest plots. Funnel plots were generated, Egger’s tests were conducted for continuous data, and Peter’s tests were conducted for dichotomous data to assess potential publication bias. A sensitivity analysis, conducted by sequentially omitting individual studies, was used to confirm the stability of the aggregated results.

Furthermore, based on results of the meta-analysis, we used the Grade of Recommendations Assessment, Development and Evaluation (GRADE) method to assess the quality of evidence for each outcome. Evidence certainty was classified into four grades: High, Moderate, Low, and Very Low. Evidence from randomized controlled trials is generally of high quality; however, its validity may be compromised by five factors: (1) Risk of bias, (2) inconsistency, (3) imprecision, (4) indirectness, and (5) publication bias. Conversely, three factors may improve its validity: (1) Large effect sizes, (2) dose-response relationships, and (3) plausible confounding. Each outcome was rated by two independent reviewers, and any disagreements were resolved through discussion or adjudication by a third researcher. The Summary of Findings Table was created using GRADEpro GDT software.

## 3 Results

### 3.1 Literature search and quality assessment

**FIGURE 1:**
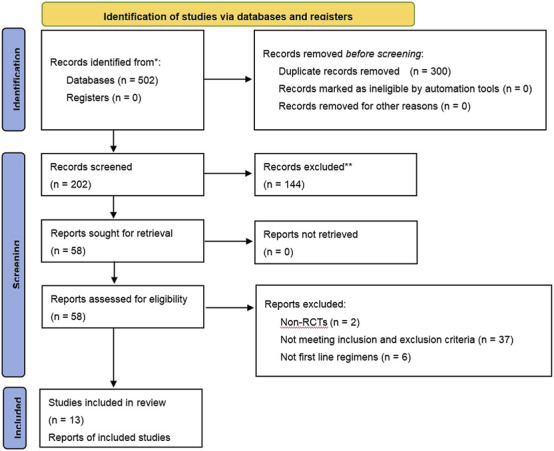
Study selection.

Of the 502 studies identified in the literature search, 300 duplicates were removed. After screening and full-text review, 13 studies (Dai, 2021; Huang et al., 2024; Jiang et al., 2019; Shi and Chen, 2013; Wang and LI, 2020; Wang et al., 2022; Yang, 2016; Yang et al., 2016; Zhou et al., 2022; Zhang et al., 2018; Zhang, 2016; Zheng et al., 2021; Zhou et al., 2017) involving 899 patients were included, encompassing six CHIs treatments: Kanglaite, Aidi, Shenfu, Shenmai, Xiaoaiping, and Elemene injection. Five types of EGFR-TKIs were investigated in 13 studies, which were categorized as first-generation (erlotinib, gefitinib, and icotinib) and third-generation (osimertinib and almonertinib). A flowchart illustrating the study selection process is depicted in Figure 1, while Table 2 enumerates the characteristics of the included studies.

**TABLE 2:** Initial features of studies included in the network meta-analysis of patients with NSCLC having advanced EGFR mutations.

Study ID	Cases (E/C)	Sex (M/F)	Age (year, E/C)	Intervention in the experimental group	Intervention in the control group	Course of treatment (Days)	Outcomes
Dai S, 2021	96 (48/48)	53/43	35–67/37–69	Gefitinib 0.25 g Qd po + KLTI 200 mL ivgtt Qd	Gefitinib 0.25 g Qd po	21d × 2	①②③④⑤⑦
Huang YH, 2024	60 (30/30)	37/23	65.00 ± 8.13/63.17 ± 8.45	Almonertinib 110 mg Qd po + SMI 60 mL ivgtt Qd	Almonertinib 110 mg Qd po	21d × 4	①②③④⑥
Jiang J, 2019	60 (28/32)	37/23	53–75/52–76	Gefitinib 0.25 g Qd po + ADI 60 mL ivgtt Qd	Gefitinib 0.25 g Qd po	15d × 2	①②
Shi QH, 2013	45 (25/20)	12/33	38–75/35–67	Gefitinib 0.25 g Qd po + KLTI 100 mL ivgtt Qd	Gefitinib 0.25 g Qd po	21d × 2	①②③
Wang F, 2020	60 (30/30)	35/25	38–77	Erlotinib 150 mg Qd po + ADI 50 mL ivgtt Qd	Erlotinib 150 mg Qd po	20d × 2	①②③④⑤⑥⑦
Wang HF, 2022	82 (41/41)	50/32	54.6 ± 7.2/55.2 ± 7.3	Icotinib 125 mg Qd po + SMI 60 mL ivgtt Qd	Icotinib 125 mg Qd po	28d × 3	①②④⑤⑥⑦
Yang WJ, 2016	86 (43/43)	47/39	36–80	Icotinib 125 mg Qd po + KLTI 200 mL ivgtt Qd	Icotinib 125 mg Qd po	21d × 3	①②③④⑤⑦
Yang WQ, 2016	64 (32/32)	33/31	40–70	Gefitinib 0.25 g Qd po + XAPI 60 mL ivgtt Qd	Gefitinib 0.25 g Qd po	28d × 2	①②④⑤⑥
Zhou J, 2022	96 (50/46)	63/33	69.38 ± 6.59/70.21 ± 5.98	Osimertinib 80 mg Qd po + SFI 60–80 mL ivgtt Qd	Osimertinib 80 mg Qd po	28d × 4	①②③⑦
Zhang LJ, 2018	62 (31,31)	36/26	22–82/20–81	Gefitinib 0.25 g Qd po + ADI 100 mL ivgtt Qd	Gefitinib 0.25 g Qd po	30d × 2	①②④⑤
Zhang QH, 2016	30 (15,15)	17/13	63.2 ± 1.7/64.6 ± 2.2	Gefitinib 0.25 g Qd po + EI 500 mg ivgtt Qd	Gefitinib 0.25 g Qd po	21d × 2	①②③⑤⑥
Zheng QH, 2021	38 (12,26)	13/25	38–80	Icotinib 125 mg Qd po + EI 500 mg ivgtt Qd	Icotinib 125 mg Qd po	21d × 2	①②④⑥
Zhou Y, 2017	120 (60,60)	72/48	66–79	Gefitinib 0.25 g Qd po + EI 500 mg ivgtt Qd	Gefitinib 0.25 g Qd po	28d	①②③⑦

KLTI, kanglaite injection; SMI, shenmai injection; ADI, aidi injection; XAPI, xiaoaiping injection; SFI, shenfu injection; EI, elemene injection; E = experimental group; C, control group; M, male; F, female; ① objective tumor response (ORR); ② disease control rate (DCR); ③ quality of life (QOL); ④ dermatologic toxicities; ⑤ gastrointestinal toxicities; ⑥ hepatic insufficiency; ⑦ immune index.

### 3.2 Evaluation of methodological quality

**FIGURE 2:**
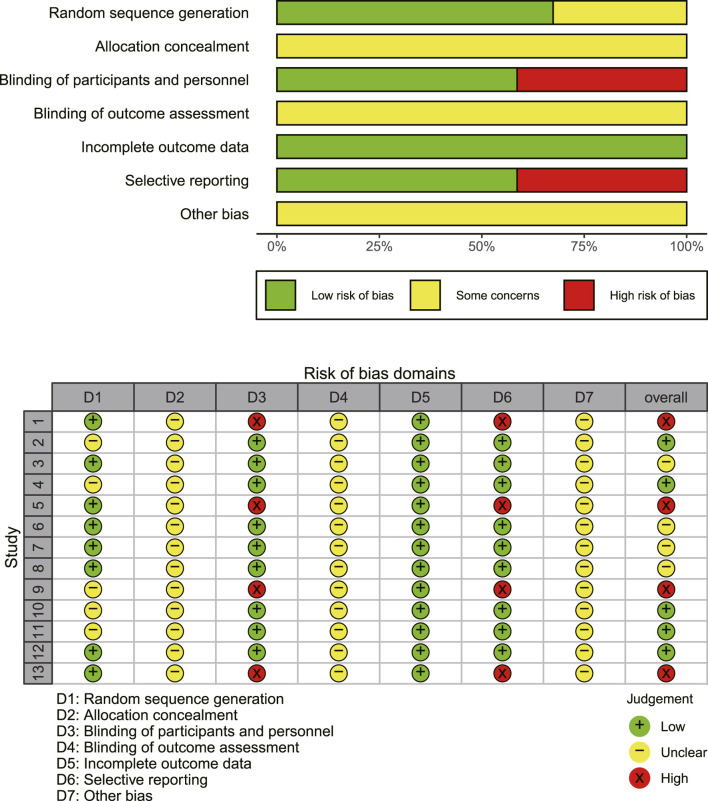
An overview of the findings from studies evaluated using the Cochrane risk of bias tool. Study numbers in the traffic plot: 1. Dai (2021), 2. Huang et al. (2024), 3. Jiang et al. (2019), 4. Shi and Chen (2013), 5. Wang and Li (2020), 6. Wang et al. (2022), 7. Yang et al. (2016), 8. Yang et al. (2016), 9. Zhou et al. (2022), 10. Zhang et al. (2018), 11. Zhang (2016), 12. Zheng et al. (2021), 13. Zhou et al. (2017).

The results of the methodological assessment are depicted in Figure 2. The case number of each study was used as a weight in the analysis. While all 13 publications referenced randomization, only eight specified the precise mechanism of random allocation (Dai, 2021; Jiang et al., 2019; Wang and LI, 2020; Wang et al., 2022; Yang, 2016; Yang et al., 2016; Zheng et al., 2021; Zhou et al., 2017) and demonstrated a low risk of random sequence generation. None of the studies specified techniques for allocation concealment, leaving the risk ambiguous. The selected studies did not reference double-blinding or blinding of outcome assessments. The outcomes, including ORR, DCR, adverse effects, and immune indexes, were considered objective and thus unaffected by double-blinding. However, EORTC QLQ-C30 was implemented as an outcome measure in four studies (Dai, 2021; Wang and LI, 2020; Zhou et al., 2022; Zhou et al., 2017), which is somewhat subjective. Consequently, these studies were classified as high risk for participant and personnel blinding. Regarding reporting bias, four studies (Dai, 2021; Wang and LI, 2020; Zhou et al., 2022; Zhou et al., 2017) using EORTC QLQ-C30 did not present complete findings of the questionnaire and were categorized as “high risk.”

### 3.3 Primary outcomes

All 13 studies reported ORR and DCR as primary outcomes, with 899 patients (445 in the experimental group and 454 in the control group). A subgroup analysis was conducted based on the use of first- or third-generation EGFR-TKIs in the control group due to the significant difference in efficacy between them (Zhao et al., 2019). Simultaneously, another subgroup was created based on the various CHIs used in the experimental group to investigate their individual effects. However, the findings from Xiaoaiping and Shenfu injection subgroups cannot be deemed credible because each CHI was represented by only one study in the subgroups.

**FIGURE 3:**
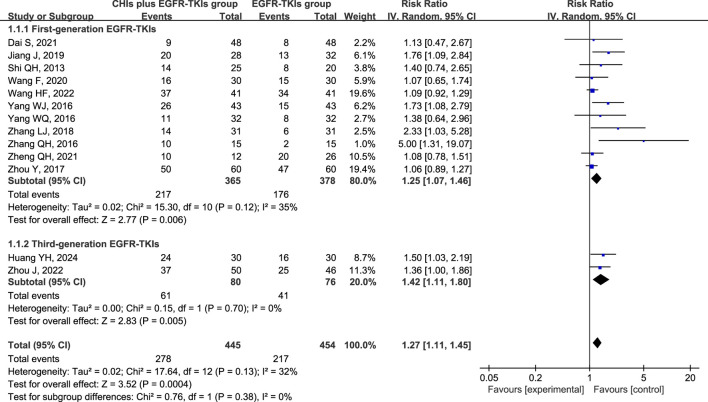
Forest plot illustrating ORR by subgroups of first- or third-generation EGFR-TKIs.

**FIGURE 4:**
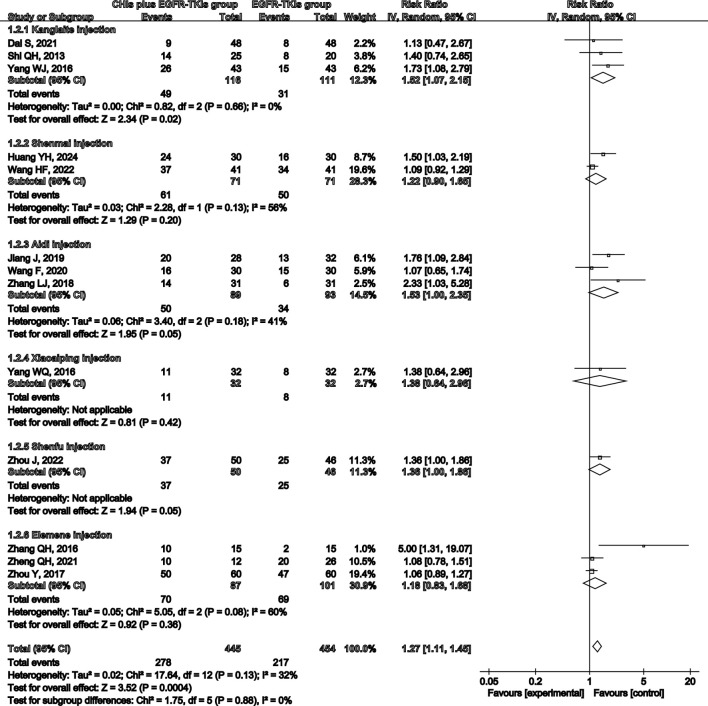
Forest plot illustrating ORR by subgroups of different types of CHIs.

Total analysis of the included papers on ORR demonstrated low heterogeneity (*p* = 0.13, I^2^ = 32%); however, there were differences in the types of CHIs and EGFR-TKIs included in the studies. The random-effects model was a safer and more conservative choice because it accounted for potential differences across studies, yielding generalizable results. Our data indicated that patients receiving CHIs + EGFR-TKIs demonstrated significantly higher ORRs than those receiving EGFR-TKIs alone (RR = 1.27, 95% CI: 1.11–1.45, *p* = 0.0004). Subgroup analysis of first- and third-generation EGFR-TKIs yielded comparable outcomes (RR = 1.25, 95% CI: 1.07–1.46, *p* = 0.006 versus RR = 1.42, 95% CI: 1.11–1.80, *p* = 0.005) (Figure 3). Additionally, Kanglaite injection significantly improved ORR in combination with EGFR-TKIs (RR = 1.52, 95% CI: 1.07–2.15, *p* = 0.02). Conversely, Shenmai injection (RR = 1.22, 95% CI: 0.90–1.65, *p* = 0.20), Aidi injection (RR = 1.53, 95% CI: 1.00–2.35, *p* = 0.05), and Elemene injection (RR = 1.18, 95% CI: 0.83–1.68, *p* = 0.36) demonstrated no significant influence (Figure 4).

**FIGURE 5:**
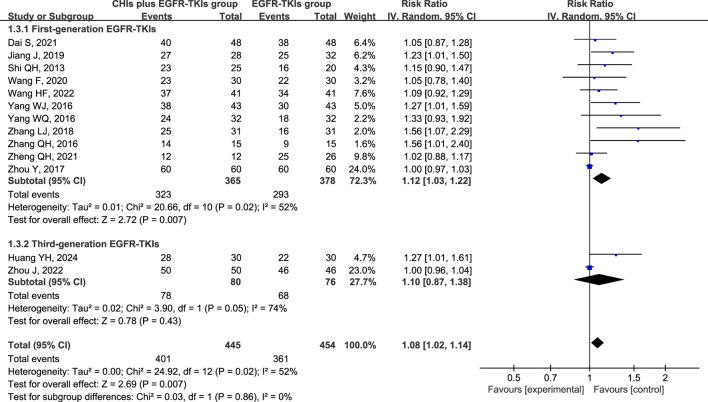
Forest plot illustrating DCR by subgroups of first- or third-generation EGFR-TKIs.

**FIGURE 6:**
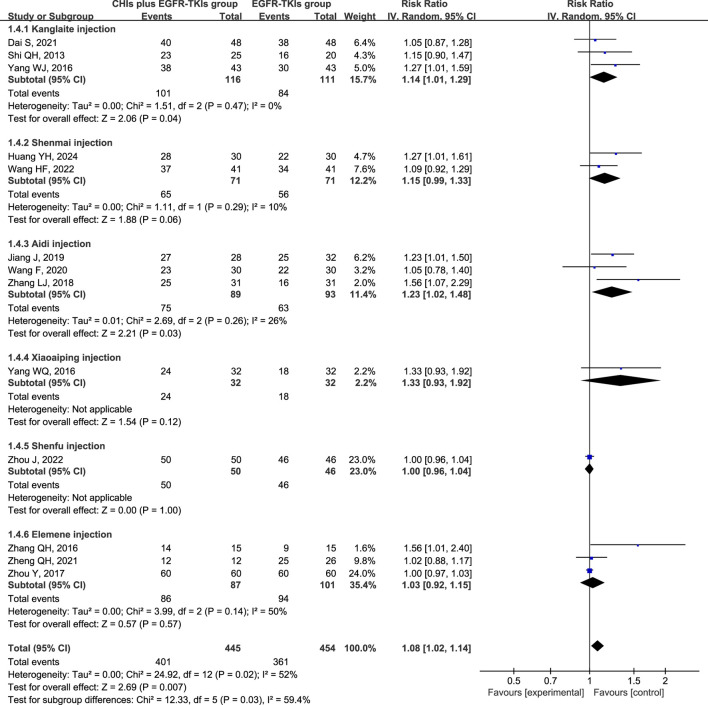
Forest plot illustrating DCR by subgroups of different types of CHIs.

Heterogeneity analysis of DCR revealed significant heterogeneity (*p* = 0.02, I^2^ = 52%), implying the appropriateness of a random-effect model. DCR was significantly improved in patients using CHIs (RR = 1.08, 95% CI: 1.02–1.14, *p* = 0.007) and in the subgroup of first-generation EGFR-TKIs (RR = 1.12, 95% CI: 1.03–1.22, *p* = 0.007). However, no significant change was observed in the third-generation EGFR-TKIs subgroup (RR = 1.10, 95% CI: 0.87–1.38, *p* = 0.43) (Figure 5). In several subgroups of different CHIs, administration of Kanglaite injection (RR = 1.14, 95% CI: 1.01–1.29, *p* = 0.04) and Aidi Injection (RR = 1.23, 95% CI: 1.02–1.48, *p* = 0.03) in conjunction with EGFR-TKIs significantly improved DCR. Shenmai (RR = 1.15, 95% CI: 0.99–1.33, *p* = 0.06) and elemene (RR = 1.03, 95% CI: 0.92–1.15, *p* = 0.57) injections demonstrated no significant influence (Figure 6).

### 3.4 Secondary outcomes

#### 3.4.1 Quality of life (QOL)

In 8 out of 13 studies, QOL was assessed using various instruments: Four used the EORTC QLG Core Questionnaire (EORTC QLQ-C30) (Dai, 2021; Wang and Li, 2020; Zhou et al., 2022; Zhou et al., 2017), three used KPS (Huang et al., 2024; Shi and Chen, 2013; Yang, 2016), and one used the Zubrod scale (ZPS) (Zhang QH, 2016). Except for Zhang QH, 2016, all studies reported a significant enhancement in QOL. However, different assessment methodologies were identified within these groups. For instance, only two of the four studies using the EORTC QLQ-C30 provided average scores for three dimensions (functional, symptom, and overall health), whereas the others only reported scores for functional areas. Two studies used changes in KPS scores to calculate QOL improvement rate using different definition methods. The significant variability in the reporting of QOL outcomes across studies indicates that both meta- and subgroup analyses were inapplicable.

#### 3.4.2 Adverse effects

**FIGURE 7:**
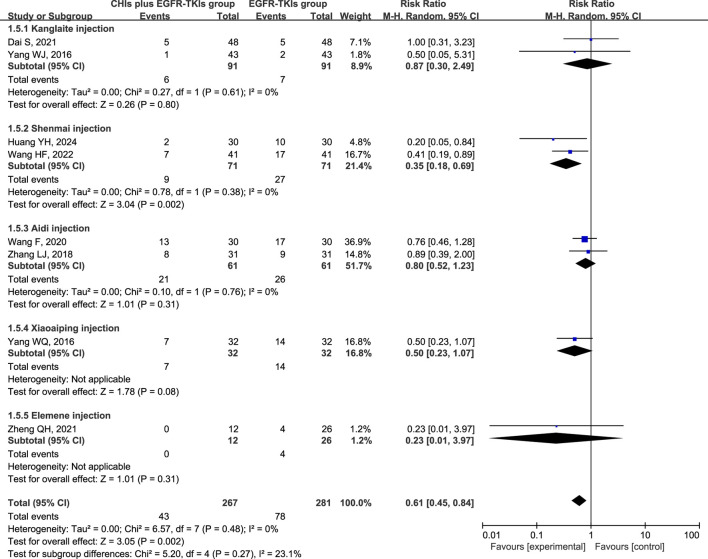
Forest plot illustrating the incidence of dermatologic toxicity in the included studies.

**FIGURE 8:**
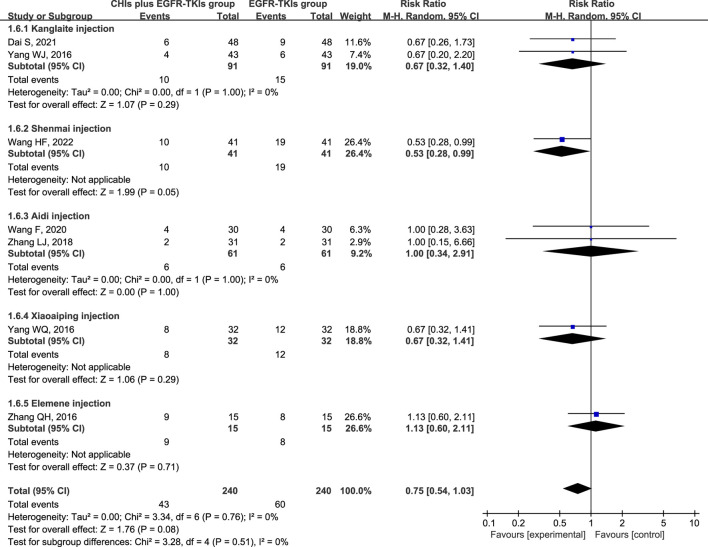
Forest plot illustrating the incidence of gastrointestinal toxicity in the included studies.

**FIGURE 9:**
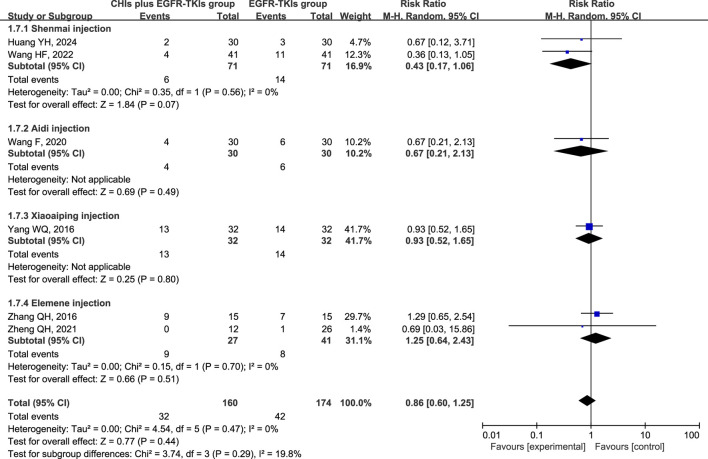
Forest plot illustrating the incidence of hepatic insufficiency in the included studies.

The three most frequently reported adverse effects in the included studies were dermatologic toxicity, gastrointestinal toxicity, and hepatic insufficiency. A random-effects model was used to assess adverse effects arising from differences in the types of CHIs and EGFR-TKIs included in the studies. The effects of each CHI were determined using a subgroup analysis. The most commonly reported adverse effect, dermatologic toxicities, was included in eight trials with 548 individuals (267 in the experimental group and 281 in the control group). The relative risks revealed that EGFR-TKIs alone were less effective than combination therapies of CHIs and EGFR-TKIs in reducing the occurrence of dermatologic toxicities (RR = 0.61, 95% CI: 0.45–0.84, *p* = 0.002). Subsequent analysis revealed that only the combination of Shenmai injection and EGFR-TKIs (RR = 0.35, 95% CI: 0.18–0.69, *p* = 0.002) significantly affected the incidence of dermatologic toxicities, whereas other subgroups demonstrated no notable impact (Figure 7). In a review of seven trials including 480 participants (240 in the experimental group and 240 in the control group), no significant reduction in the incidence of gastrointestinal toxicities was observed in the experimental group (RR = 0.75, 95% CI: 0.54–1.03, *p* = 0.08) compared to the control group. Despite a reduction in gastrointestinal toxicities among the subgroup receiving Shenmai injection (RR = 0.53, 95% CI: 0.28–0.99, *p* = 0.05), the findings were deemed inconclusive due to the inclusion of only one study (Figure 8). The meta-analysis of hepatic insufficiency (six studies) reported no significant difference between the experimental and control groups (RR = 0.86, 95% CI: 0.60–1.25, *p* = 0.47), with a total of 334 participants (160 in the experimental and 174 in the control group). Comparable results were observed across all analyzed subgroups (Figure 9).

#### 3.4.3 Immune index

**FIGURE 10:**
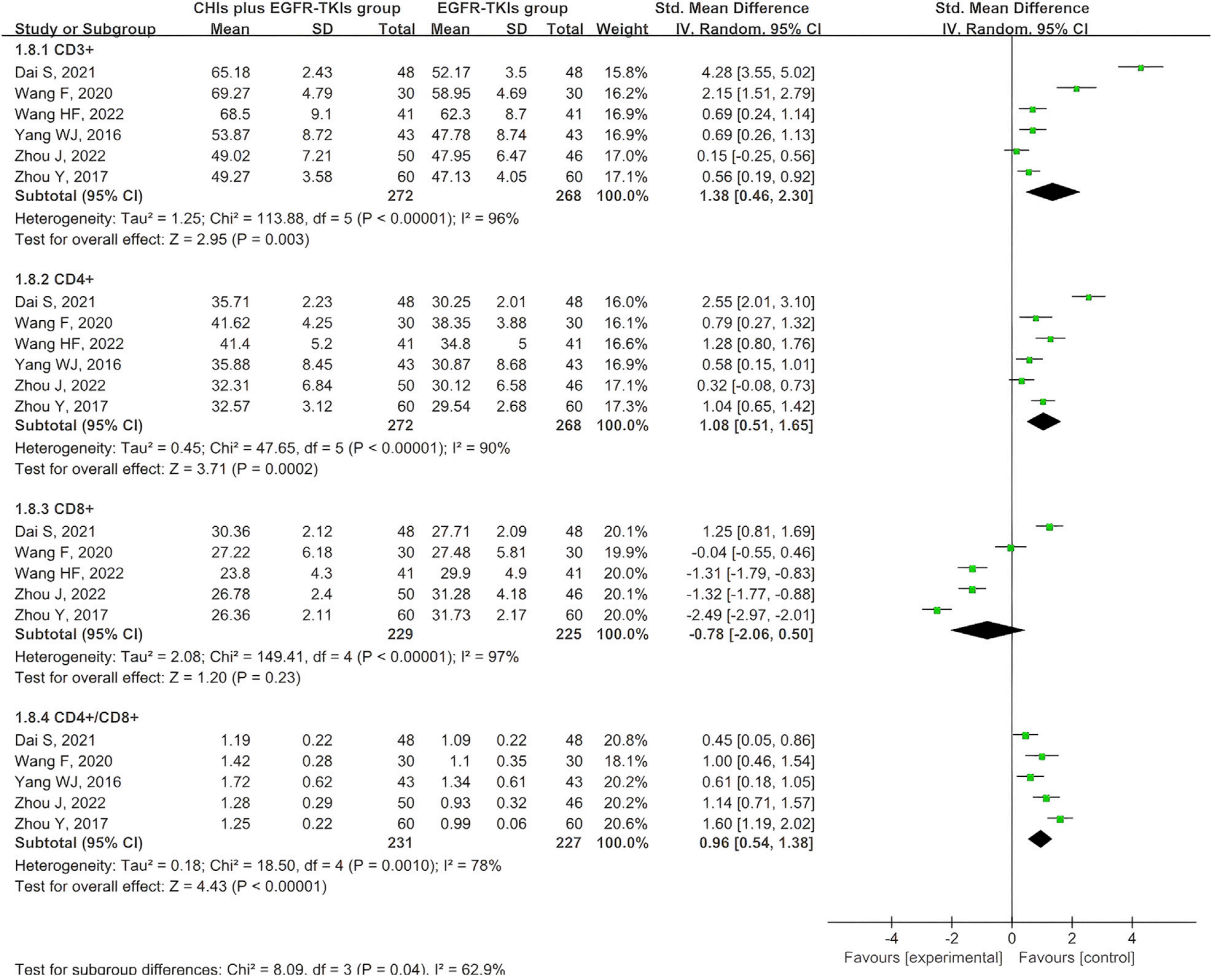
Forest plot illustrating the immune index.

Six articles documented the immune index, specifically the expression of T cell subtypes. All six studies reported CD3^+^ and CD4^+^ expression, whereas five reported CD8^+^ expression and the CD4^+^/CD8^+^ ratio. Given the variability of the four outcomes, the data were obtained using a random-effects model. The aggregated SMD analysis revealed that CHIs and EGFR-TKIs significantly enhanced the expression of CD3^+^ (SMD = 1.38, 95% CI: 0.46–2.30, *p* = 0.003), CD4^+^ (SMD = 1.08, 95% CI: 0.51–1.65, *p* = 0.0002), and the CD4^+^/CD8^+^ ratio (SMD = 0.96, 95% CI: 0.54–1.38, *p* < 0.00001) compared to EGFR-TKIs alone. However, no significant difference was observed in CD8^+^ expression between the two groups (SMD = −0.78, 95% CI: –2.06 to 0.50, *p* = 0.23) (Figure 10).

Considering the high heterogeneity in expression of CD3^+^, CD4^+^, CD8^+^, and CD4^+^/CD8^+^ ratio, we investigated potential covariates, including continuous variables such as publication year, sample size, and treatment course, as well as categorical variables such as methodological quality, EGFR-TKI types, and CHI types. Meta-regression analysis was used for continuous variables, while for categorical variables, subgroup analysis was used. Meta-regression analysis revealed no statistical association between the continuous variables and significant heterogeneity (Supplementary Table S4). Subgroup analysis of categorical variables revealed that studies with high methodological quality risk were the primary source of heterogeneity in CD3^+^ expression (Supplementary Figure S1A), as well as high heterogeneity in CD4^+^, CD8^+^, and CD4^+^/CD8^+^ ratio (Supplementary Figures S12-S4). Given the small number of studies in each subgroup, reliable subgroup analyses were not possible to assess statistical significance for different types of EGFR-TKIs and CHIs (Supplementary Figures S1-S4). Therefore, we only provided a descriptive report. The significant differences observed in outcomes across subgroups demonstrated that variations among these types of EGFR-TKIs and CHIs may be a potential source of heterogeneity.

### 3.5 Publication bias

**FIGURE 11:**
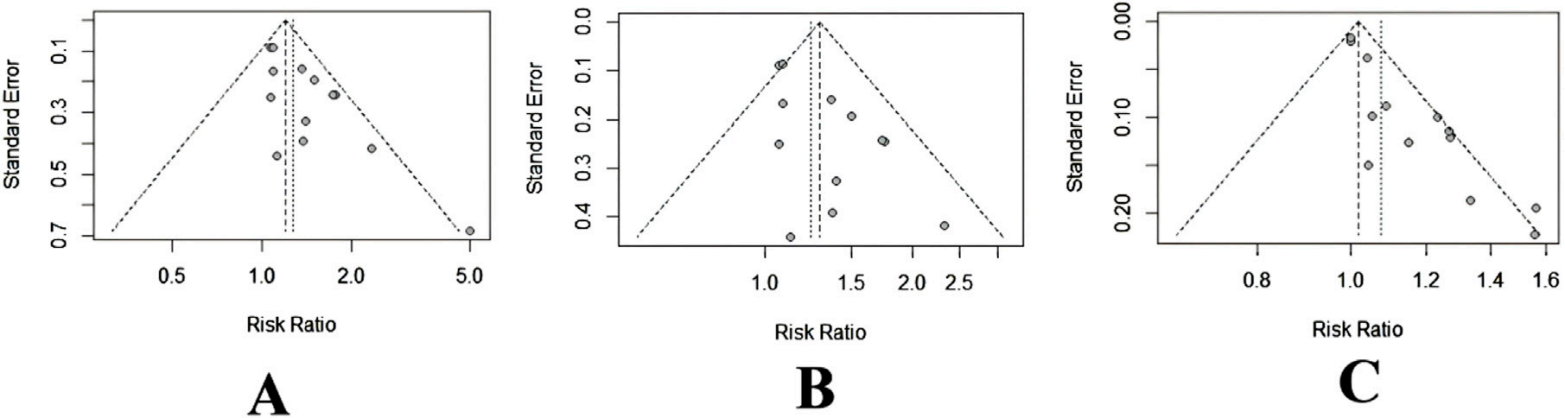
Funnel plot of ORR and DCR. **(A)** ORR. **(B)** ORR after removing Zhang (2016). **(C)** DCR.

The potential publication bias in the primary outcomes, ORR and DCR, was investigated using funnel plots (Figure 11). Furthermore, Peter’s tests were used for quantitative analysis. Funnel plots of ORR revealed a possible asymmetry due to clearly deviating spots (Figure 11A), confirmed by Peter’s tests (*p* = 0.04). This phenomenon implied that smaller-scale studies may have been published because their results achieved higher benefit estimates, whereas a few studies with smaller benefits or negative outcomes may have remained unpublished or excluded. Such publication bias may have resulted in a potential overestimation of the overall benefit. To identify the source of publication bias, the study with the most obvious heterogeneity (Zhang QH, 2016) was removed, and the analysis was repeated. The funnel plot results revealed better symmetry (Figure 11B), whereas Peter’s tests revealed no evidence of publication bias (*p* = 0.59). The eliminated studies may introduce publication bias due to their smaller sample sizes and larger effect sizes. Furthermore, Peter’s tests demonstrated no discernible bias for the DCR (*p* = 0.19), consistent with the funnel plot analysis (Figure 11C).

### 3.6 Sensitivity analysis

We performed a sensitivity analysis for outcomes by excluding studies one by one (Supplementary Figure S5). The results revealed that the combined effect size estimates were robust, thereby improving the reliability of our conclusions.

### 3.7 Certainty of evidence analysis through the GRADE method

**FIGURE 12:**
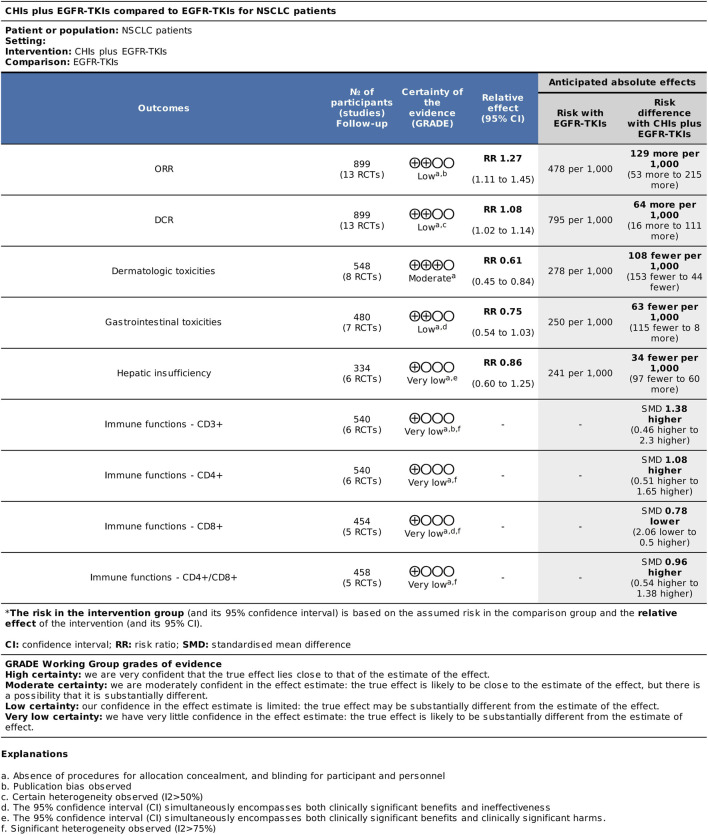
Summary of findings for certainty of evidence analysis using the GRADE method.

Based on the results of systematic reviews and meta-analyses, we conducted certainty of evidence analysis for major efficacy, adverse reaction, and immune index outcomes (Figure 12). All outcome grades were downgraded by one level due to the absence of allocation concealment and blinding procedures for participants and personnel in the inclusion study. The certainty of evidence for ORR was rated as low for major efficacy outcomes. Publication bias resulted in further downgrade. The certainty of evidence for DCR was downgraded by another level to low due to heterogeneity (I^2^ = 52%). The certainty of evidence for dermatologic toxicities was moderate. The results for gastrointestinal toxicities and hepatic insufficiency were low and very low due to imprecision. The 95% CI for gastrointestinal toxicity outcomes included both clinically significant benefits and ineffectiveness, whereas the 95% CI for hepatic insufficiency included both clinically significant benefits and harm. Moreover, the certainty of evidence for the immune index was very low due to significant heterogeneity (I^2^ > 75%), which downgraded the quality of evidence by two levels.

## 4 Discussion

### 4.1 Efficacy and safety analysis

Our systematic review included 13 studies with 899 patients. Overall, meta-analysis of ORR and DCR revealed that the combination of CHIs and EGFR-TKIs was significantly more efficacious than EGFR-TKIs alone. The funnel plot asymmetry and Peter’s test of ORR indicated a small-sample effect, potentially leading to an overestimation of ORR benefits. Therefore, we should be cautiously optimistic about the current ORR results. In terms of certainty of evidence, the combination of CHIs and EGFR-TKIs potentially improved anti-tumor efficacy with uncertain results, compared to EGFR-TKIs alone (low certainty evidence).

Subgroup analysis for first-generation EGFR-TKIs revealed that CHIs were associated with improved ORR and DCR, while in subgroup analysis for third-generation EGFR-TKIs, CHIs were associated with improved ORR without any significant influence on DCR. The subgroup analysis for third-generation EGFR-TKIs included only two studies, probably due to the delayed clinical application and research progress of third-generation EGFR-TKIs compared to first-generation EGFR-TKIs. Additionally, the differences in efficacy and adverse reactions between the two generations have expanded the opportunities for TCM interventions in combination with first-generation EGFR-TKIs. Due to the limited number of studies on third-generation EGFR-TKIs and the small sample sizes, the statistical efficacy was insufficient to reliably elucidate the real effect differences. Given their widespread use, high-quality clinical studies are required to focus on the combination of CHIs with third-generation EGFR-TKIs, which are currently scarce.

Subsequent investigation revealed that the combination of Kanglaite injection with EGFR-TKIs was more effective in improving ORR, while the combination of Kanglaite or Aidi injection with EGFR-TKIs effectively improved DCR. Kanglaite injection demonstrated numerous activities, including apoptosis induction in cancer cells, impeding cancer cell mitosis, killing malignant cells, and improving immune function in advanced NSCLC (Wen et al., 2020). A study demonstrated that Kanglaite injection decreased serum miRNA-21 expression in individuals with advanced lung cancer (Wu et al., 2018). Numerous clinical studies have demonstrated that Aidi injection affects tumor cells by slowing their proliferation, inducing apoptosis, and modulating immune function (Guo et al., 2021). The studies included in the meta-analysis used ORR and DCR as primary outcomes, with a limited follow-up period of 3–4 months. However, to validate the impact of CHIs in conjunction with EGFR-TKIs, the anti-tumor efficacy requires additional assessment over an extended observation period using PFS or overall survival.

Regarding QOL, only qualitative analysis was conducted due to the significant variability of different outcome measures. Numerous studies have validated improvements in QOL; however, the presence of fragmentary data necessitates caution in interpreting these findings. Future research must establish standardized reporting criteria for QOL and provide comprehensive data to assess the impact of CHIs on QOL.

Overall, the combination of CHIs with EGFR-TKIs was associated with reduced dermatologic toxicity (moderate certainty evidence). However, the risks of gastrointestinal toxicity and hepatic insufficiency remain uncertain (low or very low certainty evidence). The combination of Shenmai injection with EGFR-TKIs significantly reduced the incidence of dermatological toxicity compared to EGFR-TKIs alone. EGFR is often expressed in hair follicles, sebaceous glands, and keratinocytes. EGFR-TKIs may influence cellular proliferation and differentiation, induce the release of chemokines and cytokines, and facilitate the development of skin inflammation (Curry et al., 2014). Ginsenosides have been recognized as the principal active component in SMI (Liu et al., 2012). Excessive generation of reactive oxygen species (ROS) is associated with severe inflammation and cellular apoptosis in several cell types. Targeting ROS is considered a vital mechanism by which ginsenosides regulate pathological inflammation (Paik et al., 2023). Future experimental studies are needed to elucidate the involvement of CHIs in this process.

Fatigue, a common adverse effect of EGFR-TKIs, has not been reported in any of the trials reviewed thus far. The FLAURA trial data revealed that the incidence of fatigue with osimertinib therapy was 58.1% (Leighl et al., 2020). Fatigue significantly affects QOL and adversely affects patients’ adherence to therapy when severe. There is a demand for clear and effective intervention strategies. Consequently, studies on TCM interventions, including commercial Chinese polyherbal preparations, should be conducted in this regard.

This meta-analysis is the first to assess the impact of CHIs on the immune index following EGFR-TKI therapy. CHIs combined with EGFR-TKIs improved the expression of CD3^+^ and CD4^+^ T cells, as well as the CD4^+^/CD8^+^ ratio, implying that CHIs exert their anti-tumor activity by augmenting human immune function. Due to the risk of bias and significant heterogeneity, the evidence was highly uncertain. Our results should be interpreted cautiously. Future clinical trials should investigate these issues further to obtain more compelling data.

### 4.2 Limitations

This study has some limitations. First, there were several biases in the included studies. We identified publication bias based on the funnel plots about ORR, which was confirmed by Peter’s tests. Despite all studies reporting random allocation, only eight specified the exact random allocation method used. The lack of reporting on randomization methods made it impossible to confirm that all studies achieved true randomization, potentially leading to selection bias. Although the impact of such bias on the direction of combined effect sizes was difficult to quantify precisely, it undermined the reliability of our findings. This was because the observed effects might partially stem from baseline imbalances rather than the intervention. None of the studies disclosed procedures for allocation concealment, participant and personnel blinding, or rates of follow-up and withdrawal. The absence of allocation concealment indicated that researchers involved in patient enrollment might have anticipated the grouping arrangements. This could have resulted in systematic differences between the intervention and control groups at baseline, even if randomization was appropriate. Such scenarios often artificially inflate the efficacy of interventions. The absence of participant and personnel blinding may have introduced significant performance bias. Patients aware of their group assignments may have given more positive evaluations due to psychological expectations, while researchers familiar with the groups may have unconsciously favored intervention outcomes when assessing subjective indicators. Consequently, some observed inter-group differences may originate from such expectation effects rather than the biological efficacy of the interventions, potentially leading to overestimation of actual therapeutic outcomes. Furthermore, four of these studies exhibited selective reporting, indicating a degree of reporting bias. Researchers reported only indicators with statistically significant differences and ignored negative outcome indicators, especially in QOL scales that contain many items, potentially leading to an overestimation of the results. Second, linguistic bias may have influenced our results because all the relevant research was conducted in Chinese. Our findings warrant careful interpretation due to these concerns and should be validated through more comprehensive investigations with enhanced methodological rigor. Third, the lack of patient categorization by gender, smoking status, or EGFR mutation type (exon 19 deletion and Leu858Arg mutations) may influence therapy efficacy. Furthermore, because numerous studies lacked specific outcomes, they were excluded from all analyses. For instance, only eight treatments were evaluated for dermatologic toxicities, while seven studies addressed gastrointestinal toxicities and six focused on hepatic insufficiency, potentially compromising the precision of the analytical outcomes. Fourth, subgroup analysis revealed that the current clinical data for the combination of TCM injections and EGFR-TKIs are insufficient. In several subgroup analyses, only one study was included, yielding unreliable analytical outcomes.

## 5 Conclusion

Our meta-analysis revealed that combining CHIs with EGFR-TKIs improved ORR and DCR, reduced side effects, and modulated immune function in patients with NSCLC. Therefore, in conjunction with targeted therapy, CHIs may serve as a supplemental therapy for NSCLC. However, our results must be interpreted cautiously due to the generally low certainty evidence. Obtaining additional clinical data from randomized controlled trials with more stringent designs is necessary to comprehensively examine overall and individual effectiveness and safety, yielding more reliable findings for clinical applications.

## Data Availability

The raw data supporting the conclusions of this article will be made available by the authors, without undue reservation.

## Glossary

ADI: Aidi Injection
CBM: Chinese Biological Medicine Database
CHIs: Chinese herbal injections
CNKI: China National Knowledge Infrastructure
CR: complete relief
DCR: disease control rate
EGFR: epidermal growth factor receptor
EGFR-TKIs: epidermal growth factor receptor-tyrosine kinase inhibitors
EI: Elemene injection
EORTC QLQ-C30: EORTC QLG Core Questionnaire
GRADE: Grade of Recommendations AssessmentDevelopment and Evaluation
KLTI: Kanglaite injection
KPS: Karnofsky scale
MDR: multiple-drug resistance
NSCLC: non-small cell lung cancer
ORR: objective response rate
OS: overall survival
PD: progressive disease
PFS: progression free survival
PR: partial remission
PRISMA: preferred reporting items for systematic reviews and meta-analyses
QOL: quality of life
RCTs: Randomized controlled trials
RECIST: Response Evaluation Criteria in Solid Tumors
RRs: risk ratios
SD: stable disease
SFI: Shenfu injection
SMD: Standardized mean differences
SMI: Shenmai injection
TCM: Traditional Chinese medicine
XAPI: Xiaoaiping injection
