# Supplementary material for: Chinese herbal injections combined with EGFR-TKIs for intervention of non-small cell lung cancer: a systematic review and meta-analysis
Source: Front Pharmacol. 2025 Nov 19;16:1670501. doi: 10.3389/fphar.2025.1670501 (PMC12672443; doi:10.3389/fphar.2025.1670501)
Supplement: Supplementary file 1 [file Supplementaryfile1.docx]

**Supplementary material content**

**Table S1.** PRISMA 2020 checklist.

**Table S2.** Basic information of included commercial Chinese polyherbal preparation.

**Table S3.** Literature search strategy of Chinese and English databases.

**Table S4.** Meta-regression analysis results for expression of CD3^+^, CD4^+^, CD8^+^ and CD4^+^/CD8^+^ ratio.

**Figure S1 A-C.** Subgroup analysis of expression of CD3^+^ based on categorical variables.

**Figure S2 A-C.** Subgroup analysis of expression of CD4^+^ based on categorical variables.

**Figure S3 A-C.** Subgroup analysis of expression of CD8^+^ based on categorical variables.

**Figure S4 A-C.** Subgroup analysis of CD4^+^/CD8^+^ ratio based on categorical variables.

**Figure S5 A-I.** Sensitivity analysis through exclusion of studies one-by-one.

**Table S1.** PRISMA 2020 checklist

| **Section and Topic** | **Item #** | **Checklist item** | **Location where item is reported** |
| --- | --- | --- | --- |
| **TITLE** | | |  |
| Title | 1 | Identify the report as a systematic review. | Title |
| **ABSTRACT** | | |  |
| Abstract | 2 | See the PRISMA 2020 for Abstracts checklist. | Abstract |
| **INTRODUCTION** | | |  |
| Rationale | 3 | Describe the rationale for the review in the context of existing knowledge. | 1 Introduction |
| Objectives | 4 | Provide an explicit statement of the objective(s) or question(s) the review addresses. | 1 Introduction |
| **METHODS** | | |  |
| Eligibility criteria | 5 | Specify the inclusion and exclusion criteria for the review and how studies were grouped for the syntheses. | 2.3 Study selection |
| Information sources | 6 | Specify all databases, registers, websites, organisations, reference lists and other sources searched or consulted to identify studies. Specify the date when each source was last searched or consulted. | 2.2 Data sources and searches |
| Search strategy | 7 | Present the full search strategies for all databases, registers and websites, including any filters and limits used. | 2.2 Data sources and searches |
| Selection process | 8 | Specify the methods used to decide whether a study met the inclusion criteria of the review, including how many reviewers screened each record and each report retrieved, whether they worked independently, and if applicable, details of automation tools used in the process. | 2.5 Data obtaining and quality evaluation |
| Data collection process | 9 | Specify the methods used to collect data from reports, including how many reviewers collected data from each report, whether they worked independently, any processes for obtaining or confirming data from study investigators, and if applicable, details of automation tools used in the process. | 2.5 Data obtaining and quality evaluation |
| Data items | 10a | List and define all outcomes for which data were sought. Specify whether all results that were compatible with each outcome domain in each study were sought (e.g. for all measures, time points, analyses), and if not, the methods used to decide which results to collect. | 2.4 Outcome indicators and evaluation criteria |
|  | 10b | List and define all other variables for which data were sought (e.g. participant and intervention characteristics, funding sources). Describe any assumptions made about any missing or unclear information. | 2.4 Outcome indicators and evaluation criteria |
| Study risk of bias assessment | 11 | Specify the methods used to assess risk of bias in the included studies, including details of the tool(s) used, how many reviewers assessed each study and whether they worked independently, and if applicable, details of automation tools used in the process. | 2.5 Data obtaining and quality evaluation |
| Effect measures | 12 | Specify for each outcome the effect measure(s) (e.g. risk ratio, mean difference) used in the synthesis or presentation of results. | 2.6 Statistical analysis |
| Synthesis methods | 13a | Describe the processes used to decide which studies were eligible for each synthesis (e.g. tabulating the study intervention characteristics and comparing against the planned groups for each synthesis (item #5)). | 2.3 Study selection |
|  | 13b | Describe any methods required to prepare the data for presentation or synthesis, such as handling of missing summary statistics, or data conversions. | 2.5 Data obtaining and quality evaluation |
|  | 13c | Describe any methods used to tabulate or visually display results of individual studies and syntheses. | 2.6 Statistical analysis |
|  | 13d | Describe any methods used to synthesize results and provide a rationale for the choice(s). If meta-analysis was performed, describe the model(s), method(s) to identify the presence and extent of statistical heterogeneity, and software package(s) used. | 2.6 Statistical analysis |
|  | 13e | Describe any methods used to explore possible causes of heterogeneity among study results (e.g. subgroup analysis, meta-regression). | 2.6 Statistical analysis |
|  | 13f | Describe any sensitivity analyses conducted to assess robustness of the synthesized results. | 2.6 Statistical analysis |
| Reporting bias assessment | 14 | Describe any methods used to assess risk of bias due to missing results in a synthesis (arising from reporting biases). | 2.5 Data obtaining and quality evaluation |
| Certainty assessment | 15 | Describe any methods used to assess certainty (or confidence) in the body of evidence for an outcome. | 2.6 Statistical analysis |
| **RESULTS** | | |  |
| Study selection | 16a | Describe the results of the search and selection process, from the number of records identified in the search to the number of studies included in the review, ideally using a flow diagram. | Figure 1 |
|  | 16b | Cite studies that might appear to meet the inclusion criteria, but which were excluded, and explain why they were excluded. | 3.1 Literature search and quality assessment |
| Study characteristics | 17 | Cite each included study and present its characteristics. | Table 2 |
| Risk of bias in studies | 18 | Present assessments of risk of bias for each included study. | Figure 2 |
| Results of individual studies | 19 | For all outcomes, present, for each study: (a) summary statistics for each group (where appropriate) and (b) an effect estimate and its precision (e.g. confidence/credible interval), ideally using structured tables or plots. | Table 2 |
| Results of syntheses | 20a | For each synthesis, briefly summarise the characteristics and risk of bias among contributing studies. | 3.2 Evaluation of methodological quality |
|  | 20b | Present results of all statistical syntheses conducted. If meta-analysis was done, present for each the summary estimate and its precision (e.g. confidence/credible interval) and measures of statistical heterogeneity. If comparing groups, describe the direction of the effect. | 3 RESULTS |
|  | 20c | Present results of all investigations of possible causes of heterogeneity among study results. | 3 RESULTS |
|  | 20d | Present results of all sensitivity analyses conducted to assess the robustness of the synthesized results. | 3.6 Sensitivity analysis |
| Reporting biases | 21 | Present assessments of risk of bias due to missing results (arising from reporting biases) for each synthesis assessed. | 3.5 Publication bias |
| Certainty of evidence | 22 | Present assessments of certainty (or confidence) in the body of evidence for each outcome assessed. | 3.7 Certainty of evidence analysis through the GRADE method |
| **DISCUSSION** | | |  |
| Discussion | 23a | Provide a general interpretation of the results in the context of other evidence. | 4.1 Efficacy and safety analysis |
|  | 23b | Discuss any limitations of the evidence included in the review. | 4.2 Limitations |
|  | 23c | Discuss any limitations of the review processes used. | 4.2 Limitations |
|  | 23d | Discuss implications of the results for practice, policy, and future research. | 4.1 Efficacy and safety analysis |
| **OTHER INFORMATION** | | |  |
| Registration and protocol | 24a | Provide registration information for the review, including register name and registration number, or state that the review was not registered. | 2 Methods |
|  | 24b | Indicate where the review protocol can be accessed, or state that a protocol was not prepared. | 2 Methods |
|  | 24c | Describe and explain any amendments to information provided at registration or in the protocol. | NA |
| Support | 25 | Describe sources of financial or non-financial support for the review, and the role of the funders or sponsors in the review. | Funding |
| Competing interests | 26 | Declare any competing interests of review authors. | NA |
| Availability of data, code and other materials | 27 | Report which of the following are publicly available and where they can be found: template data collection forms; data extracted from included studies; data used for all analyses; analytic code; any other materials used in the review. | Supplementary Material |

**Table S2.** Basic information of included commercial Chinese polyherbal preparation.

| **Pharmacopeial Drug Name** | **Composition** | **Implementation standards** | **Batch numbers** |
| --- | --- | --- | --- |
| Kanglaite injection | Yiyiren | China Food and Drug Administration National Drug Standard WS3-301 (Z-038)-2006(Z)-2013 | Z10970091 |
| Aidi injection | Banmao, Renshen, Huangqi, Ciwujia | China Food and Drug Administration National Drug Standard WS3-B-3809-99-2002 | Z52020236 |
| Shenmai injection | Hongshen, Maidong | China Food and Drug Administration National Drug Standard WS3-B-3428-98-2010 | Z33020019 |
| Shenfu injection | Hongshen, Heifupian | China Food and Drug Administration National Drug Standard WS3-B-3427-98-2013 | Z51020664 |
| Xiaoaiping injection | Tongguanteng | China Food and Drug Administration National Drug Standard WS-10630 (ZD-0630)-2002-2013Z-2019 | Z20025868 |
| Elemene injection | Wenyujin | China Food and Drug Administration National Drug Standard WS1-(X-095)-2000Z-2017 | H10960114 |
| **Indications** | | | |
| Kanglaite injection | This product is suitable for primary non-small cell lung cancer and primary liver cancer with qi-yin deficiency and spleen deficiency-dampness trapping that are not suitable for surgery. It has a certain efficacy when combined with radiotherapy and chemotherapy. It can also have a certain anti-illness quality effect and pain relief effect for middle and advanced stage tumor patients. | | |
| Aidi injection | This product is used for primary liver cancer, lung cancer, rectal cancer, malignant lymphoma, gynecological malignancies, etc. | | |
| Shenmai injection | This product is used to treat shock, coronary heart disease, viral myocarditis, chronic pulmonary heart disease and granulocytopenia of qi-yin deficiency type. It can improve the immune function of cancer patients, and has a certain effect of enhancing efficacy when used with chemotherapy drugs, and can reduce the toxic and adverse reactions caused by chemotherapy drugs. | | |
| Shenfu injection | This product is used for yang qi sudden loss of collapse (infectious, hemorrhagic, fluid loss shock, etc.); it can also be used for yang deficiency (qi deficiency) caused by palpitation, restlessness, wheezing, stomach pain, diarrhea, arthralgia, etc. | | |
| Xiaoaiping injection | This product is used for esophageal cancer, stomach cancer, lung cancer, liver cancer, and can be combined with radiotherapy, chemotherapy adjuvant therapy. | | |
| Elemene injection | This product combined with conventional chemotherapy and radiotherapy can enhance the efficacy of lung cancer, liver cancer, esophageal cancer, nasopharyngeal carcinoma, brain tumor, bone metastatic cancer and other malignant tumors, and reduce the toxic side effects of radiotherapy and chemotherapy. It can also be used for interventional therapy, intracavitary chemotherapy and cancerous pleural effusion and abdominal fluid. | | |
| **Pharmacological effects** | | | |
| Kanglaite injection | 1. This product demonstrates inhibitory effects against Lewis lung carcinoma in mice, B16 melanoma lung metastases, W256 carcinosarcoma in rats, and human liver cancer QGY transplanted into nude mice. 2. When combined with low-dose cyclophosphamide, it enhances the inhibitory effect on W256 carcinosarcoma in rat xenografts. It also suppresses leukopenia (elevated ALT) caused by 5-fluorouracil, cyclophosphamide, or cisplatin in mice, as well as elevated blood urea nitrogen (BUN) induced by cisplatin. 3. The product promotes spleen lymphocyte proliferation in tumor-bearing mice, enhances NK cell activity, and improves macrophage phagocytic function. It prolongs both normobaric hypoxia tolerance time and swimming duration in both tumor-bearing and normal mice.   4. The product inhibits acetic acid-induced pain responses in mice, reducing writhing frequency. | | |
| Aidi injection | Aidi injection demonstrates significant inhibitory effects on S180, H22, and EAC solid tumors in mice. It enhances both non-specific and specific immune functions while boosting the body's stress response capacity. When combined with anticancer drugs 5-FU and CTX or used in conjunction with radiotherapy, it exhibits synergistic efficacy that maintains white blood cell and platelet counts within normal ranges. In vitro tumor inhibition experiments reveal this formulation exhibits direct cytotoxicity and growth suppression against cancer cells. | | |
| Shenmai injection | NA | | |
| Shenfu injection | NA | | |
| Xiaoaiping injection | NA | | |
| Elemene injection | The primary biological activities involve inhibiting tumor cell mitosis, inducing apoptosis, and suppressing tumor growth. Pharmacological studies demonstrate that intraperitoneal injection of oleanolic acid emulsion significantly suppresses DNA, RNA, and protein synthesis in tumor cells. The agent directly affects cell membranes, causing tumor cell rupture while enhancing their immunogenicity to stimulate immune responses. With minimal toxicity, it shows negligible impact on normal cells and peripheral leukocytes. The intravenous LD50 is (270.07±18.93) mg/kg, while oral LD50 exceeds 5 g/kg. Common doses show no teratogenic or mutagenic effects in mice. | | |
| **Side effects** | | | |
| Kanglaite injection | Clinical cases of fat allergy are occasionally observed, such as chills, fever, mild nausea, and reversible elevation of liver transaminases. These symptoms usually resolve spontaneously and the patient adapts after 3–5 days of use. Mild phlebitis is also occasionally seen | | |
| Aidi injection | When this product is used for the first time, some patients occasionally experience reactions such as facial flushing, urticaria, and fever. A very small number of patients may also have palpitations, chest tightness, and nausea. | | |
| Shenmai injection | Urticarial rash, facial flushing, chest tightness, palpitations, generalized weakness, numbness, dizziness, headache, anaphylactic shock, grand mal seizure, nausea, vomiting, jaundice, gastrointestinal bleeding, acute liver and kidney function impairment, tachycardia, angina pectoris, and phlebitis. | | |
| Shenfu injection | Allergic reactions: May manifest as itching, rash, allergic dermatitis, pale complexion, chest tightness, breathing difficulty, laryngeal edema, palpitations, cyanosis, blood pressure decrease, etc. In severe cases, anaphylactic shock may occur.  Systemic reactions: Including chills, fever, fatigue, profuse sweating, back pain, and so on.  Nervous system damage: Dizziness, headache, insomnia, tremor, convulsions, numbness of lips and limbs, etc.  Cardiovascular system: Facial flushing, palpitations, chest tightness, tachycardia, arrhythmia, fluctuating blood pressure, etc.  Digestive system damage: Nausea, vomiting, abdominal distension, abdominal pain, diarrhea, hiccups, dry mouth, gastric discomfort, abnormal liver function, etc.  Respiratory system damage: Cyanosis of lips, cough, dyspnea, rapid breathing, etc.  Urinary system damage: Urinary retention, edema, etc.  Others: Nosebleed, redness/swelling/pain at the injection site, phlebitis, visual abnormalities, etc | | |
| Xiaoaiping injection | Allergic reactions: generalized skin flushing, rash, itching, dyspnea (difficulty breathing), palpitations, cyanosis, hypotension (decrease in blood pressure), laryngeal edema, anaphylactic shock, etc.  Musculoskeletal: migratory muscle pain, joint pain, etc.  Systemic reactions: fever, chills, pain, fatigue, etc.  Skin and appendages: rash, itching, excessive sweating, etc.  Digestive system: nausea, vomiting, abdominal pain, diarrhea, etc.  Respiratory system: difficulty breathing, cough, etc.  Cardiovascular system: chest tightness, palpitations, increased or decreased blood pressure, etc.  Nervous system: dizziness, headache, etc.  Others: pain at injection site, phlebitis, etc. | | |
| Elemene injection | 1. Systemic abnormalities: chills, fever, shivering, sweating, chest discomfort, chest pain, back pain, generalized pain, swelling, fatigue, etc.; 2. injection site reactions: pain, swelling, erythema, induration at the injection site; 3. Vascular and lymphatic abnormalities: phlebitis, erythema, etc.; 4. Gastrointestinal system abnormalities: nausea, vomiting, abdominal discomfort, pain, bloating, diarrhea, etc.; 5. Skin and subcutaneous tissue manifestations: rashes (including urticaria, maculopapular eruptions, pruritic rashes, papules, erythematous eruptions), itching, erythema, skin edema, skin reactions, etc.; 6. Respiratory system abnormalities: dyspnea, shortness of breath, suffocation sensation, coughing, etc.; 7. Neurological abnormalities: dizziness, headache, paresthesia, tremors, etc.; 8. Immune system abnormalities: hypersensitivity reactions, anaphylaxis, anaphylactic shock, etc.; 9. Cardiac abnormalities: palpitations, cardiac palpitations, etc.; 10. Laboratory abnormalities: elevated or decreased blood pressure, reduced oxygen saturation, etc.;   11. Notable adverse reactions: Allergic reactions: This product may cause allergic reactions, with severe cases leading to anaphylactic shock. Symptoms may include chest tightness, chest pain, chills, sweating, erythema, cyanosis, rashes, urticaria, pruritus, palpitations, dyspnea, etc. | | |
| **Interactions** | | | |
| Kanglaite injection | Not suitable for mixing with other drugs | | |
| Aidi injection | Be cautious of additive toxicities (e.g., myelosuppression, hepatorenal toxicity) when combined with chemotherapeutics like Cisplatin or Cyclophosphamide | | |
| Shenmai injection | Incompatible with: Veratrum nigrum, Faeces Trogopterori, Goderol injection, and antibiotics (especially penicillins) | | |
| Shenfu injection | This product should not be directly mixed or used in combination with Coenzyme A, Vitamin K3, Aminophylline, Doxorubicin Hydrochloride, Danshen injection, Omeprazole Sodium for injection, or Cerebroprotein Hydrolysate injection.  If this product needs to be used together with traditional Chinese medicines such as Pinellia, Trichosanthes Fruit, Fritillaria, Bai Lian (Bletilla), Bai Ji, Wu Ling Zhi (Trogopterus dung), or Veratrum, please consult a physician. | | |
| Xiaoaiping injection | Contraindicated to mix with other drugs in the same container | | |
| Elemene injection | There is synergistic effect when used in combination with radiotherapy or other chemotherapeutic drugs and biological response regulators, and synergistic effect when combined with heating therapy. | | |
| **Identification** | | | |
| Kanglaite injection | Take 5 ml of the sample and heat in a water bath to allow phase separation. Collect the oil layer and prepare a solution with petroleum ether (60–90℃) to a concentration of 40 mg/ml.  Reference Solution: Prepare a reference solution of Coix seed oil in the same manner, making a 40 mg/ml solution in petroleum ether.  Thin Layer Chromatography Conditions:  Plate: Silica gel G  Developing solvent: Petroleum ether–diethyl ether–acetic acid (9:1:0.1)  Visualization: Spray with 5% vanillin–sulfuric acid solution and heat at 105°C for 5 minutes.  Judgment Criteria:  In the chromatogram of the test solution, spots of the same color (typically purplish red or blue-purple) should appear at the same positions as those in the chromatogram of the reference solution. | | |
| Aidi injection | 1. Take 50 ml of this product, concentrate to approximately 10 ml, add 50 ml of ethanol, mix well, and filter. Wash the precipitate twice with ethanol. Dissolve the precipitate in 5 ml of distilled water, take 1 ml and place it in a test tube. Warm gently, then add 5 drops of 5% α-naphthol ethanol solution, shake well, and slowly add 0.5 ml of concentrated sulfuric acid along the wall of the tube. At the interface of the two liquids, a purplish-red ring appears.  2. Take 50 ml of this product, place in a separatory funnel, and extract twice with 30 ml each time of water-saturated n-butanol. Combine the extracts, evaporate to dryness in a water bath, dissolve the residue in 3 ml of distilled water, and apply it to a previously prepared DA-201 resin column (inner diameter 1–1.5 cm, length 15 cm; packed with 12 cm DA-201 resin, topped with 2 g neutral alumina). Wash the column with 100 ml water, then elute with 50 ml of 40% methanol. Collect the eluate, evaporate to dryness, and dissolve the residue in 1 ml methanol to obtain the test solution.  Separately, prepare a mixed reference solution by dissolving ginsenosides Re, Rg₁, Rb, and astragaloside in methanol to give a concentration of 1 mg/ml for each component. According to the Thin Layer Chromatography method (Appendix VI B), spot 5 μl each of the above two solutions on the same silica gel G plate. Use the lower layer of chloroform–ethyl acetate–methanol–water (4:8:3:4), after leaving at below 10°C, as the developing solvent. Develop, remove, dry, spray with 10% ethanolic sulfuric acid solution, and heat at 105°C for several minutes. Observe under UV lamp (365 nm). In the test sample chromatogram, spots of the same fluorescent color as the reference sample should appear at the corresponding positions. | | |
| Shenmai injection | Take 5 ml of the sample, evaporate to dryness in a water bath, then dissolve the residue in 1 ml of ethanol to prepare the test solution. Separately, take reference standards of ginsenoside Rb1, Rg1, and Re, and dissolve in ethanol to prepare a mixed solution containing 2 mg of each per 1 ml, to be used as the reference solution. According to the Thin Layer Chromatography method (Appendix VI B of the Chinese Pharmacopoeia, 2010 Edition, Volume I), spot 2–5 μl each of the test solution and the reference solution on the same silica gel GF254 plate. Use the lower layer (after phase separation below 10°C) of chloroform:methanol:water (13:7:2) as the developing solvent. Develop, remove, dry, spray with 10% ethanolic sulfuric acid solution, and heat at 105°C until the spots are clearly visible. Inspect under UV light at 365 nm. In the chromatogram of the test solution, spots of the same color will appear at positions corresponding to those of the reference standard.  Take 40 ml of the sample and add 3 ml of hydrochloric acid. Heat in a water bath for 1 hour, cool, then extract with 40 ml of diethyl ether by shaking. Evaporate the ether layer to dryness and dissolve the residue in 1 ml of chloroform to prepare the test solution. Separately, take 2 g of Ophiopogon japonicus (Mai Dong) reference medicinal material, decoct with water for 30 minutes, filter, concentrate the filtrate to about 40 ml, and prepare the reference solution in the same manner. According to the Thin Layer Chromatography method (Appendix VI B of the Chinese Pharmacopoeia, 2010 Edition, Volume I), spot 5–10 μl each of the test solution and the reference solution on the same silica gel G plate. Use dichloromethane:acetone (4:1) as the developing solvent. Develop, remove, dry, spray with 10% ethanolic sulfuric acid solution, and heat at 105°C until the spots are clearly visible. In the chromatogram of the test solution, spots of the same color will appear at positions corresponding to those of the reference medicinal material.  Take 1 ml of the sample, dilute to about 10 ml with 50% acetonitrile, and mix well to prepare the test solution. Separately, take an appropriate amount of Shenmai reference extract and make a solution containing 2 mg per 1 ml with 50% acetonitrile as the reference extract solution. According to the High Performance Liquid Chromatography (HPLC) method (Appendix VI D of the Chinese Pharmacopoeia, 2010 Edition, Volume I), use an Alltech PrevailTM Carbohydrate ES column as the stationary phase, acetonitrile:water (80:20) as the mobile phase, and use an evaporative light scattering detector for detection. Inject 10 μl each of the test solution and the reference extract solution into the chromatograph. In the chromatogram of the test solution, four major peaks should be observed at the same retention times as those in the reference extract. | | |
| Shenfu injection | 1. Identification Reaction:  Take 1 ml of the sample, place it in an evaporating dish, and evaporate to dryness in a water bath. Dissolve the residue with 0.5 ml acetic anhydride, transfer to a test tube, and carefully add 0.5 ml sulfuric acid along the tube wall. A brownish-red ring appears at the interface of the two liquids.  2. Thin Layer Chromatography (TLC) Identification:  Take 30 ml of the sample, place it in a separatory funnel, add 30 ml chloroform, shake well, and allow to stand. Collect the upper layer, evaporate to dryness, dissolve the residue in 2 ml water, add 10 ml water-saturated n-butanol, and sonicate for 30 minutes. Collect the supernatant, add 3 times its volume of ammonia solution, shake well, and allow to separate layers. Collect the upper layer and evaporate to dryness; dissolve the residue in 1 ml methanol as the test solution.  Separately, prepare a reference herbal material solution: Take 1 g of red ginseng as reference material, add 30 ml ethanol, reflux in a water bath for 30 minutes, filter, evaporate the filtrate to dryness, dissolve the residue in 30 ml water, filter, and process the filtrate in the same manner as above to obtain the reference herbal material solution.  Prepare a mixed reference solution of ginsenosides Rb1, Re, and Rg1 in methanol, each at 2 mg/ml.  According to the method for Thin Layer Chromatography (Appendix VI B), spot 10 μl of the test solution, 20 μl of the reference herbal solution, and 2 μl of the mixed reference solution onto the same silica gel G TLC plate. Develop with chloroform–ethyl acetate–methanol–water (15:40:22:10, use the lower layer stored at below 10°C) as the developing solvent. Remove and dry the plate, spray with 10% ethanolic sulfuric acid solution, and heat at 105°C until spots are clearly visible.  In the chromatogram of the test solution, spots of identical color appear at the corresponding positions as those in the chromatogram of the reference herbal material and the reference substance. | | |
| Xiaoaiping injection | Take 10 ml of this product, add water to make a total of 20 ml, then add 1 ml of concentrated ammonia solution. Wash twice with 20 ml of chloroform each time by shaking; discard the chloroform layers. Wash the remaining aqueous layer twice with 15 ml of n-butanol each time by shaking; discard the n-butanol layers. Concentrate the aqueous layer to dryness and dissolve the residue in 1 ml of methanol. Allow to stand, then use the supernatant as the test solution.  Separately, take 2 g of Ventilago (Tongguan Teng) reference medicinal material, add 50 ml of water, soak for 24 hours, treat with ultrasound for 20 minutes, then filter. Concentrate the filtrate to 20 ml and prepare the reference solution in the same manner.  Following the Thin Layer Chromatography method (Appendix VI B of the Chinese Pharmacopoeia, 2000 Edition, Volume I), spot 5 µl each of the above two solutions on the same silica gel GF254 plate. Use chloroform–acetone–formic acid (15:3:2) as the developing solvent. Develop, remove, dry, and observe under a UV lamp (254 nm). In the chromatogram of the test solution, spots of the same color appear at the corresponding positions as in the chromatogram of the reference medicinal material. | | |
| Elemene injection | 1. Take 0.5ml of the product, place it in a test tube, add 1ml of ethanol, shake well, then slowly add 0.5ml of 1% vanillin sulfate solution along the wall of the tube. A purple-red ring appears at the junction of the two liquid layers, which turns purplish-purple after shaking.   2. Take 5ml of the product, place it in a separatory funnel, add 10ml of petroleum ether, shake vigorously, separate the layers, and divide the petroleum ether layer. Evaporate the petroleum ether to approximately 2.5ml to prepare the test sample solution. Separately, take an appropriate amount of β-limonene reference substance, add petroleum ether to prepare a solution containing 10mg per 1ml as the reference solution. Perform the thin-layer chromatography (Appendix V-B, Part II, Chinese Pharmacopoeia 1995 Edition) test. Accurately draw 10 μl from each of the above solutions and spot them on the same silica gel G thin-layer plate. Using petroleum ether as the developing agent, develop the spots, dry them, and spray with 1% vanillin sulfate solution. The color and position of the main spot in the test sample solution should match those in the reference solution. | | |
| **Quality control tests** | | | |
| Kanglaite injection | pH Value: 4.8–6.8 (measured by potentiometry to ensure stability)$8$.  Particle Size Distribution (Key Index):  Tested by laser particle size analyzer; particles ≤2 μm should account for ≥95%; no particles above 5 μm should be detected (to prevent vascular embolism).  Total Residue: 0.130–0.150 g/ml (determined by weighing after evaporation in a water bath, controls excipient residues).  2. Safety Tests  Heavy Metals: ≤5 ppm (lead equivalent, atomic absorption spectrophotometry).  Arsenic Salts: ≤2 ppm (Gutzeit method).  Bacterial Endotoxin: ≤1.5 EU/ml (Limulus Amebocyte Lysate test, ensures absence of pyrogen).  Sterility: In compliance with regulations (membrane filtration method, to prevent microbial contamination).  Ignited Residue: ≤0.1% (controls inorganic impurities). | | |
| Aidi injection | pH Value: Should be 3.8–5.0 (Appendix VII G).  Heavy Metals: Accurately measure 2 ml of the sample, place in a crucible, and evaporate to dryness in a water bath. Test according to the second method in Appendix IX E; heavy metals content should not exceed 0.0005 (five parts per million).  Pyrogen Test: Test as prescribed (Appendix VIII A); dilute the sample injection 10 times to the clinical concentration with corresponding pyrogen-free injection solution, and inject 3 ml per kg rabbit body weight. The result should meet requirements.  Others: Should comply with all relevant requirements for injection preparations (Appendix IU). | | |
| Shenmai injection | pH Value: Should be 5.0–6.5 (Chinese Pharmacopoeia 2010 Edition, Volume I, Appendix VIII G).  Solution Color: Accurately measure 1 ml of the product, place in a 25 ml Nessler colorimeter tube with a 10 ml marking, dilute with water to 10 ml, mix well, and compare with Yellow No. 7 Standard Color Solution (Chinese Pharmacopoeia 2010 Edition, Volume I, Appendix XIA, Method 1); the solution must not be darker.  Ignited Residue: Accurately measure 2 ml of the product, evaporate to dryness, and conduct the test as prescribed (Chinese Pharmacopoeia 2010 Edition, Volume I, Appendix XJ); not more than 1.0% (g/ml); for specifications 6 and 7, not more than 1.5% (g/ml).  Total Solids: Accurately measure 10 ml of the product, place in a previously dried evaporating dish, evaporate on a water bath to dryness, dry at 105°C for 3 hours, cool in a desiccator for 30 minutes, then promptly and accurately weigh; calculate the total solids content.  Total Solids Content: Should be 2.0%–3.5% (g/ml); for specifications 6 and 7, should be 2.5%–4.0% (g/ml).  Related Substances: Except for tannins, other related substances should comply with requirements (Chinese Pharmacopoeia 2010 Edition, Volume I, Appendix IX S).  Sterility Test: Test as prescribed; should comply with requirements (Chinese Pharmacopoeia 2010 Edition, Volume I, Appendix XIII B).  Pyrogen Test: Test as prescribed (Chinese Pharmacopoeia 2010 Edition, Volume I, Appendix XIII A); administer 2.5 ml per kg rabbit body weight, should comply with requirements.  Abnormal Toxicity: Test as prescribed (Chinese Pharmacopoeia 2010 Edition, Volume I, Appendix XVIII B, Abnormal Toxicity Test); administer by intravenous injection, should comply with requirements.  Hemolysis and Agglutination: Test as prescribed (Chinese Pharmacopoeia 2010 Edition, Volume I, Appendix XVIII B, Hemolysis and Agglutination Test); should comply with requirements.  Allergic Reaction: Test as prescribed (Chinese Pharmacopoeia 2010 Edition, Volume I, Appendix XVIII B, Allergic Reaction Test); should comply with requirements.  Osmolality: Required for specifications 6 and 7; test as prescribed (Chinese Pharmacopoeia 2010 Edition, Volume II, Appendix IX G); should be 280–320 mOsmol/kg.  Others: Should comply with all relevant requirements for injection preparations (Chinese Pharmacopoeia 2010 Edition, Volume I, Appendix IU). | | |
| Shenfu injection | pH Value: 6.8–7.2 (measured directly)  Ignition Residue: Not more than 0.2%  Hemolysis Test: Negative (General Rule 1122)  Pyrogen: Rabbit method: not more than 0.6°C/rabbit  Bacterial Endotoxin: Not more than 0.25 EU/mL  Aconitum Alkaloids Limit: Ester-type alkaloids: not more than 0.01 μg/mL (by HPLC-MS/MS) | | |
| Xiaoaiping injection | pH Value: 6.5–7.5 (measured directly)  Ignition Residue: Not more than 0.3%  Hemolysis Test: Negative (rabbit erythrocyte method, General Rule 1122)  Pyrogen: Rabbit method: ≤0.6 °C per rabbit (General Rule 1142)  Bacterial Endotoxin: Not more than 0.5 EU/ml (Limulus reagent method, General Rule 1143) | | |
| Elemene injection | 1. pH value: It should be 5.5-7.0 (Appendix VI H, Part II of the Chinese Pharmacopoeia 1995 Edition). 2. Particle size: Take one vial of this product, shake well, then measure 2ml and add 2ml of glycerol solution (1→2) and shake again. Place a drop on a slide, cover with a glass slide, and immediately examine two slides under a 15×40 microscope. Check 20 fields to ensure uniform particles without agglomeration. No particles exceeding 15μm in diameter shall be detected. If particles larger than 15μm are found, retest once and ensure compliance.   3. Pyrogens: Precisely take 2ml of this product, dilute with 5% glucose injection to 10ml, and perform testing according to the regulations (Appendix XI D, Part II of the Chinese Pharmacopoeia 1995 Edition). Administer 10ml slowly per 1kg of rabbit body weight, ensuring compliance. Other requirements: Except for clarity testing, all specifications under the injectable section shall be met (Appendix I B, Part II of the Chinese Pharmacopoeia 1995 Edition). | | |
| **Assay** | | | |
| Kanglaite injection | Accurately measure 10 mL of the product and transfer it to a 250 mL conical flask. The flask is heated to 105-110°C until complete emulsion breakdown (white emulsion disappears) occurs, then cooled. Precisely add 25 mL of 0.5 mol/L potassium hydroxide ethanol titrant solution. After heating for 30 minutes, rinse the condenser tube's inner wall and lower stopper with 10 mL of ethanol. Allow to cool, then add 5 drops of phenolphthalein indicator. Titrate with 0.5 mol/L hydrochloric acid until the pink color just fades completely. Heat to boiling; if pink reappears, continue titration until pink fades completely. Perform a blank test simultaneously. Each mL of 0.5 mol/L potassium hydroxide ethanol titrant solution is equivalent to 145.16mg triglycerides. The calculated result is multiplied by 0.8588 to obtain the final value. The product containing Job's tears oil should contain 90.0%-110.0% of the labeled amount. | | |
| Aidi injection | 1. Ginsenoside Re:  (1) Preparation of Control Solution: Weigh 8mg of ginsenoside Re control substance accurately, place it in a 5 mL volumetric flask, add methanol to dissolve and dilute to mark. Shake well to obtain solution (containing Re1.6mg of ginsenoside per 1 mL).  (2) Standard Curve Preparation: Precisely measure 0, 20,40,60,80, and 100 μl of control solution, respectively. Place each volume in a 10 mL stoppered test tube. Heat in an electric bath to evaporate the solvent, then immediately remove and cool. Add 0.2 mL of 5% vanillin-concentrated acetic acid solution and 0.8 mL of perchloric acid. Shake well and heat in a 60°℃ water bath for 15 minutes. Remove and cool with water for 2 minutes. Add 5 mL of acetic acid, shake again, and follow the spectrophotometric method (Appendix V-B) to measure absorbance at 544 nm. Plot the standard curve using absorbance as the vertical axis and concentration as the horizontal axis.  (3) Preparation of Test Solution: Accurately measure 20 mL of the product. Place in a separatory funnel and extract three times with chloroform (20 mL each). Combine the chloroform extracts. Wash twice with distilled water (5 mL each), discard chloroform. Combine the washes with the previous aqueous layer. Place in a separatory funnel and extract three times with water-saturated n-butanol (50 mL each). Combine the n-butanol extracts, add 3g of anhydrous sodium sulfate, stir until clear.  (4) Determination Method: Precisely measure 100 μL of the test solution and perform absorption determination according to the method specified in the preparation section of the standard curve, starting from "place in a 10 mL stoppered test tube". Read the absorbance value from the standard curve to determine the weight of ginsenoside Re (C48H82O18) in the test solution in μg. Calculate the total content.  (5) Each vial of this product contains no less than 2.0mg of ginsenoside Re per ginseng.  2. Sparganin: Determine by gas chromatography (Appendix VI E).  (1) Chromatographic Conditions and Systematic Applicability Test: Use polyethylene glycol 20M and methyl silicone rubber (SE-30) as stationary phases with concentrations of 10% and 5% respectively, mixed in a 1:1 ratio. Column temperature set at 180±10℃°C. Theoretical plate count calculated based on sparganin peaks should not be less than 1500.  (2) Preparation of Reference Solution: Weigh an appropriate amount of sparganin reference substance, accurately weigh it, and dissolve in chloroform to prepare a solution containing 0.1mg per mL as the reference solution.  (3) Preparation of Test Sample Solution: Weigh 50 mL of the test product and add 5 mL of 1.8 mol/L sulfuric acid solution. Perform three chloroform shake-extraction cycles (50 mL, 30 mL, 30 mL). Combine the chloroform extracts and concentrate using a K-D concentrator to adjust volume to 5 mL.  (4) Determination Method: Precipitate 5 μL of both reference standard solution and test sample solution into gas chromatographs for analysis.  (5) Each test product contains 0.008-0.030mg of spirotetramine (C10H12O4). | | |
| Shenmai injection | Total Saponins: Preparation of the Control Solution Take an appropriate amount of ginsenoside Re control substance, accurately weigh it, and dissolve in methanol to prepare a solution containing 2mg per 1 ml.   1. Standard Curve Preparation: Precisely measure 10μl, 20μl, 40μl, 60μl, 80μl, and 100μl of the control solution, respectively. Place them in 10ml stoppered test tubes, evaporate the solvent completely, then add 1ml of a mixed solution of 5% vanillin glacial acetic acid and perchloric acid (2:8) (prepared on-site as needed). Heat the mixture in a 60℃°C water bath for 15 minutes, remove, cool in an ice bath, add 5ml of glacial acetic acid, shake well, and immediately measure the absorbance at 544nm wavelength according to the UV-VIS spectrophotometric method (Appendix V-A, Part I of the 2010 Edition of the <China> Pharmacopoeia of China). Plot the standard curve using absorbance as the vertical axis and concentration as the horizontal axis. 2. Determination Method: Precisely measure 1ml of the product and add it to a pretreated macroporous resin column (D10 column 1.5cm × 12 cm). First elute with 25ml of water, discard the supernatant. Then elute with 60ml of 75% alcohol, collect the eluate, evaporate, dissolve the residue in ethanol, and transfer to a 10ml volumetric flask. Dilute with ethanol to the mark, shake well, and prepare the test solution. Precisely measure 1ml of the solution, place it in a stoppered test tube, evaporate, and follow the method described in the standard curve preparation section starting from "precisely adding 1ml of mixed solution of 5% vanillin glacial acetic acid and perchloric acid (2:8)". Measure the absorbance according to the procedure, read the concentration of the test solution from the standard curve, and calculate the final concentration. 3. The total saponins in this product, calculated as ginsenoside Re (C48H82O18), should be 0.80-2.00mg per 1ml. | | |
| Shenfu injection | 1. Preparation of Control Solution: Weigh ginsenoside Rb1 reference substance accurately and prepare a 1ml solution containing 3 mg of ginsenoside Rb1 using methanol. 2. Standard Curve Preparation: Precisely transfer 20,30,40,50, and 60 μl of the control solution into stoppered test tubes. Evaporate the solvent by water bath, cool, then add 0.2ml of 5% vanillin-concentrated glacial acetic acid solution and 0.8ml of perchloric acid per tube. Shake well, heat in a 60℃°C water bath for 15 minutes, remove, cool with ice water bath, add 5ml of glacial acetic acid, shake again. Plot the absorbance at 550nm (wavelength) against concentration using the standard curve (Appendix VＢ). 3. Method: Accurately transfer 10ml of the product to a separatory funnel. Extract four times with chloroform (10ml each), discard chloroform layer. Extract aqueous layer four times with saturated n-butanol (10ml each), combine n-butanol extracts, wash twice with saturated n-butanol (10ml each), discard water. Dry residue on water bath, dissolve in methanol, transfer to 5ml volumetric flask, dilute to mark, shake well. Prepare blank control using 10ml of 0.2% polysorbate 80 solution processed similarly. Precisely transfer 50 μl of the test solution into a stoppered test tube. Perform absorption determination according to the "evaporating solvent by water bath" method in the standard curve preparation section, calculate results.   4. The total ginsenoside of each 1ml of this product shall be no less than 0.5mg based on ginsenoside Rb1 (C54H92O23). | | |
| Xiaoaiping injection | 1. Preparation of the reference solution: Take an appropriate amount of chlorogenic acid reference substance, accurately weigh it, and add ethanol to prepare a solution containing 80 µg per 1 ml. 2. Preparation of the test solution: Take the contents specified under the content variation section of this product, mix thoroughly, accurately measure 2 ml, add ethanol to make up to 25 ml, shake well, then take 2 ml, add ethanol to make up to 25 ml, shake well, and obtain the solution. 3. Determination method: Accurately aspirate 2ml of the test solution, reference solution, and ethanol respectively, place them in 25ml stoppered graduated test tubes, add anhydrous ethanol to make up to 5 ml, then precisely add 2ml of 0.3% sodium dodecyl sulfate solution and 2ml of 0.5% potassium ferricyanide-1% ferric chloride (1:1) mixed solution respectively. Shake well, let stand in darkness for 5 minutes, add 0.1 mol/L hydrochloric acid solution to make up to 25 ml, shake well, and let stand in darkness for 20 minutes. Using the ethanol tube as a blank, measure the absorbance at 764nm wavelength according to spectrophotometry (Appendix VB of the Chinese Pharmacopoeia 2000 Edition Part I), and calculate the result.   4. The total phenolic acid content in this product per 1 ml, calculated as chlorogenic acid (C16H18O9), should be 9.0mg-13.0mg. | | |
| Elemene injection | The determination was performed by gas chromatography (Appendix V E, Part II of the 1995 Edition of the Chinese Pharmacopoeia).   1. Chromatographic conditions and system suitability test: Polyethylene glycol 20M was used as the stationary phase with a coating concentration of 10%, white diatomaceous earth carrier (60-80 mesh), and column temperature of 135-140℃. The theoretical plate count calculated based on the β-limonene peak should not be less than 1000. 2. Calibration factor determination: An appropriate amount of n-hexanol was precisely weighed and mixed with ethanol to prepare a solution containing approximately 9ml per 1ml, which was shaken evenly as an internal standard solution. A β-limonene reference substance of about 15mg was precisely weighed, placed in a 10ml volumetric flask, and 1ml of the internal standard solution was carefully added. The mixture was diluted with ethanol to the mark and shaken evenly. 1μl of the mixture was injected into the gas chromatograph to calculate the calibration factor.   3. Determination method: An appropriate amount of the product (approximately equivalent to 15mg of β-limonene) was precisely measured, placed in a 10ml volumetric flask, and 1ml of the internal standard solution was carefully added. The mixture was diluted with ethanol to the mark and shaken evenly. 1μl of the mixture was injected into the gas chromatograph. The chromatogram was recorded, and the peaks at approximately 0.6 times the relative retention time calculated for β-limonene (δ-limonene) and approximately 1.2 times the relative retention time (γ-limonene) were identified as the δ-limonene peak and γ-limonene peak, respectively. The sum of β-, γ-, and δ-limonene was calculated to obtain the final result. | | |
|  |  | | |

Note: The above information is from China Pharmaceutical Information Query Platform (recognised by the State Drug Administration of China) and the National Medical Products Administration (NMPA) of China

**Table S3.** Literature search strategy of Chinese and English databases.

| **Databases** | **Search strategy** |
| --- | --- |
| China National Knowledge Infrastructure  (CNKI) | (TKA= '肺癌' OR TKA= '肺恶性肿瘤' OR TKA= '非小细胞肺癌' OR TKA= '肺腺癌' OR TKA= '肺积' OR TKA= '息贲') AND (TKA='egfr-tkis' OR TKA= '表皮生长因子受体酪氨酸激酶抑制剂' OR TKA= '奥希替尼' OR TKA= '吉非替尼' OR TKA= '厄洛替尼' OR TKA= '埃克替尼' OR TKA= '阿法替尼') AND (TKA= '中药注射液' OR TKA= '注射液' OR TKA= '注射剂') |
| Chinese Biological Medicine Database  (CBM) | ("肺癌"[常用字段:智能] OR "肺恶性肿瘤"[常用字段:智能] OR "非小细胞肺癌"[常用字段:智能] OR "肺腺癌"[常用字段:智能] OR "肺积"[常用字段:智能] OR "息贲"[常用字段:智能]) AND ("egfr-tki"[常用字段:智能] OR "表皮生长因子受体酪氨酸激酶抑制剂"[常用字段:智能] OR "奥希替尼"[常用字段:智能] OR "吉非替尼"[常用字段:智能] OR "厄洛替尼"[常用字段:智能] OR "埃克替尼"[常用字段:智能] AND "阿法替尼"[常用字段:智能]) AND ("中药注射液"[常用字段:智能] OR "注射液"[常用字段:智能] OR "注射剂"[常用字段:智能]) |
| Wanfang Database | 主题:(肺癌 or 肺恶性肿瘤 or 非小细胞肺癌 or 肺腺癌 or 肺积 or 息贲) and 主题:(egfr-tki or 表皮生长因子受体酪氨酸激酶抑制剂 or 奥希替尼 or 吉非替尼 or 厄洛替尼 or 埃克替尼 or 阿法替尼) and 主题:(中药注射液 or 注射液 or 注射剂) |
| VIP Database for Chinese Technical Periodicals | M=(肺癌 OR 肺恶性肿瘤 OR 非小细胞肺癌 OR 肺腺癌 OR 肺积 OR 息贲) AND M=(egfr-tki OR 表皮生长因子受体酪氨酸激酶抑制剂 OR 奥希替尼 OR 吉非替尼 OR 厄洛替尼 OR 埃克替尼 OR 阿法替尼) AND M=(中药注射液 OR 注射液 OR 注射剂) |
| MEDLINE  (via PubMed) | (((((((((((((non-small-cell lung cancer[title] OR non-small cell lung cancer[title]) OR non small-cell lung cancer[title]) OR non small cell lung cancer[title]) OR non-small-cell lung carcinoma[title]) OR non-small cell lung carcinoma[title]) OR non small-cell lung carcinoma[title]) OR non small cell lung carcinoma[title]) OR nsclc[title]) AND (epidermal growth factor receptor[title/abstract] OR EGFR[title/abstract]) AND (((((((((((((((treatment[title/abstract] OR therapy[title/abstract]) OR tyrosine kinase inhibitor[title/abstract]) OR TKI[title/abstract]) OR osimertinib[title/abstract]) OR dacomitinib[title/abstract]) OR afatinib[title/abstract]) OR erlotinib[title/abstract]) OR gefitinib[title/abstract]) OR icotinib[title/abstract]) OR almonertinib[title/abstract]) OR furmonertinib[title/abstract]) OR first-line[title/abstract]) OR first line[title/abstract]) OR treatment-naive[title/abstract]) OR treatmentnaive[title/abstract]) AND (traditional Chinese medicine injection[title/abstract] OR Chinese herbal injection[title/abstract]) AND ((((((Randomized Controlled Trial[title/abstract] OR controlled clinical trial[title/abstract]) OR randomized[title/abstract]) OR randomised[title/abstract]) OR randomly[title/abstract]) OR trial[title/abstract]) OR phase[title/abstract])) AND (English[Language])) AND ("0001/01/01"[Date - Publication] : "2024/10/10"[Date - Publication]) |
| Embase  (via Ovid) | #1 'non-small-cell lung cancer' OR 'non-small cell lung cancer' OR 'non small-cell lung cancer' OR 'non small cell lung cancer' OR 'non-small-cell lung carcinoma' OR 'non-small cell lung carcinoma' OR 'non small-cell lung carcinoma' OR 'non small cell lung carcinoma' OR 'nsclc'  #2 'epidermal growth factor receptor' OR 'EGFR'  #3 'tyrosine kinase inhibitor' OR 'TKI' OR 'osimertinib' OR 'dacomitinib' OR 'afatinib' OR 'erlotinib' OR 'gefitinib' OR 'icotinib' OR 'almonertinib' OR 'furmonertinib' OR 'first-line' OR 'first line' OR 'treatment-naive' OR 'treatmentnaive'  #4 'traditional Chinese medicine injection' OR 'Chinese herbal injection'  #5 'Randomized Controlled Trial' OR 'controlled clinical trial' OR 'randomized' OR ''randomised' OR 'randomly' OR 'trial' OR 'phase'  #6 #1 AND #2 AND #3 AND #4 AND #5 |
| Cochrane Library | #1 MeSH descriptor: [Lung Neoplasms] explode all trees  #2 'non-small-cell lung cancer' OR 'non-small cell lung cancer' OR 'non small-cell lung cancer' OR 'non small cell lung cancer' OR 'non-small-cell lung carcinoma' OR 'non-small cell lung carcinoma' OR 'non small-cell lung carcinoma' OR 'non small cell lung carcinoma' OR 'nsclc'  #3 #1 or #2  #4 'epidermal growth factor receptor' OR 'EGFR'  #5 'tyrosine kinase inhibitor' OR 'TKI' OR 'osimertinib' OR 'dacomitinib' OR 'afatinib' OR 'erlotinib' OR 'gefitinib' OR 'icotinib' OR 'almonertinib' OR 'furmonertinib' OR 'first-line' OR 'first line' OR 'treatment-naive' OR 'treatmentnaive'  #6 'traditional Chinese medicine injection' OR 'Chinese herbal injection'  #7 MeSH descriptor: [Randomized Controlled Trial] explode all trees  #8 "controlled clinical trial" OR "randomized" OR "placebo" OR "drug therapy" OR "randomly" OR "trial" OR "groups"  #9 #7 or #8  #10 #3 and #4 and #5 and #6 and #9 |

| **Meta regression results for expression of CD3^+^** | | | | |
| --- | --- | --- | --- | --- |
| **Covariate** | **Estimate** | **SE** | **95%CI** | **P-value** |
| **Year** | 0.1279 | 0.2902 | (-0.4408, 0.6967) | 0.6593 |
| **Sample Size** | -0.0153 | 0.0381 | (-0.0900, 0.0594) | 0.6878 |
| **Treatment course** | -0.0242 | 0.0209 | (-0.0652, 0.0168) | 0.2469 |
| **Meta regression results for expression of CD4+** | | | | |
| **Covariate** | **Estimate** | **SE** | **95%CI** | **P-value** |
| **Year** | 0.0731 | 0.1457 | (-0.2125, 0.3586) | 0.6159 |
| **Sample Size** | 0.0058 | 0.0195 | (-0.0323, 0.0440) | 0.7640 |
| **Treatment course** | -0.0109 | 0.0109 | (-0.0322, 0.0104) | 0.3159 |
| **Meta regression results for expression of CD8+** | | | | |
| **Covariate** | **Estimate** | **SE** | **95%CI** | **P-value** |
| **Year** | 0.2923 | 0.3610 | (-0.4152, 0.9998) | 0.4181 |
| **Sample Size** | -0.0312 | 0.0330 | (-0.0960, 0.0336) | 0.3449 |
| **Treatment course** | -0.0075 | 0.0229 | (-0.0524, 0.0374) | 0.7439 |
| **Meta regression results for of CD4+/CD8+ ratio** | | | | |
| **Covariate** | **Estimate** | **SE** | **95%CI** | **P-value** |
| **Year** | -0.0272 | 0.1021 | (-0.2272, 0.1729) | 0.7900 |
| **Sample Size** | 0.0101 | 0.0111 | (-0.0116, 0.0319) | 0.3619 |
| **Treatment course** | -0.0009 | 0.0081 | (-0.0167, 0.0149) | 0.9122 |

**Table S4.** Meta-regression analysis results for expression of CD3^+^, CD4^+^, CD8^+^ and CD4^+^/CD8^+^ ratio.

**
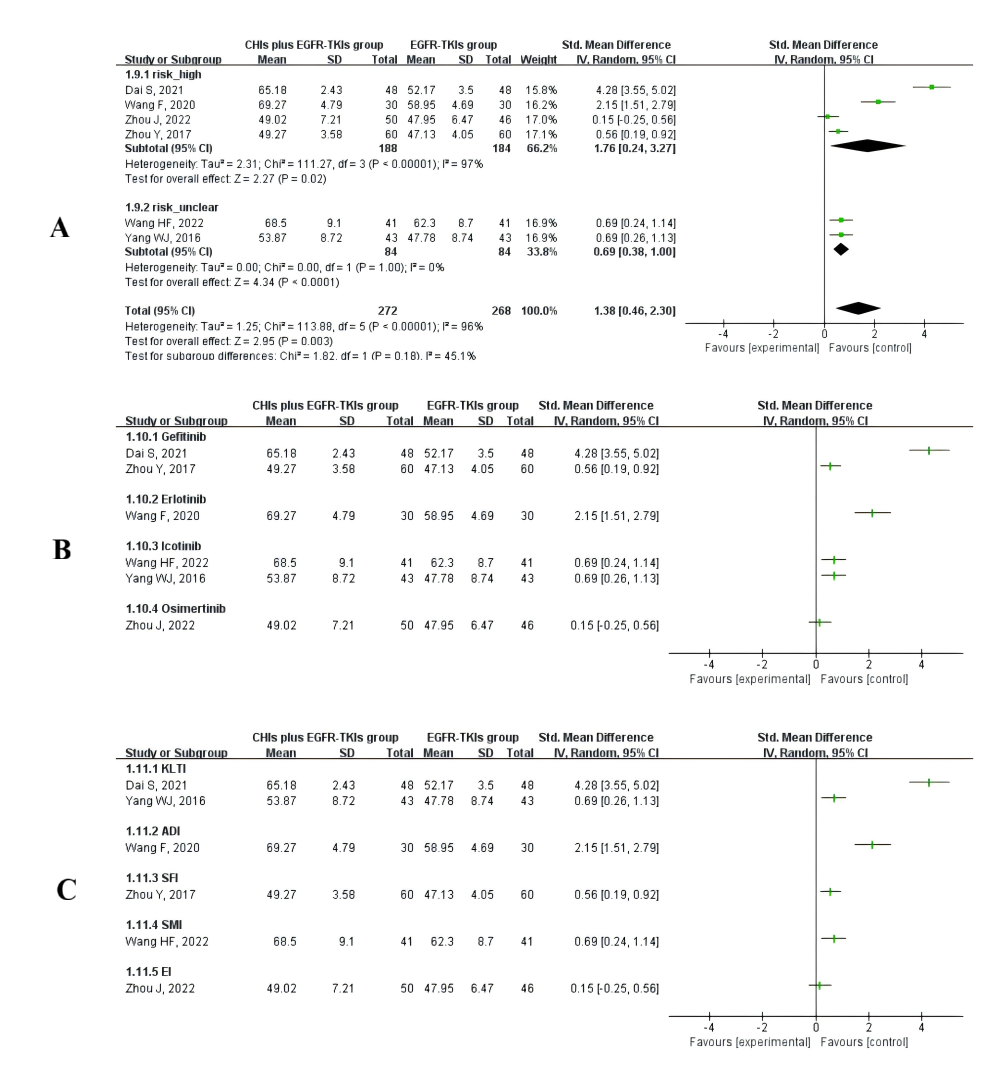
**

**Figure S1 A-C. Subgroup analysis of expression of CD3^+^ based on categorical variables.** A: methodological quality; B: EGFR-TKIs types; C: CHIs types.


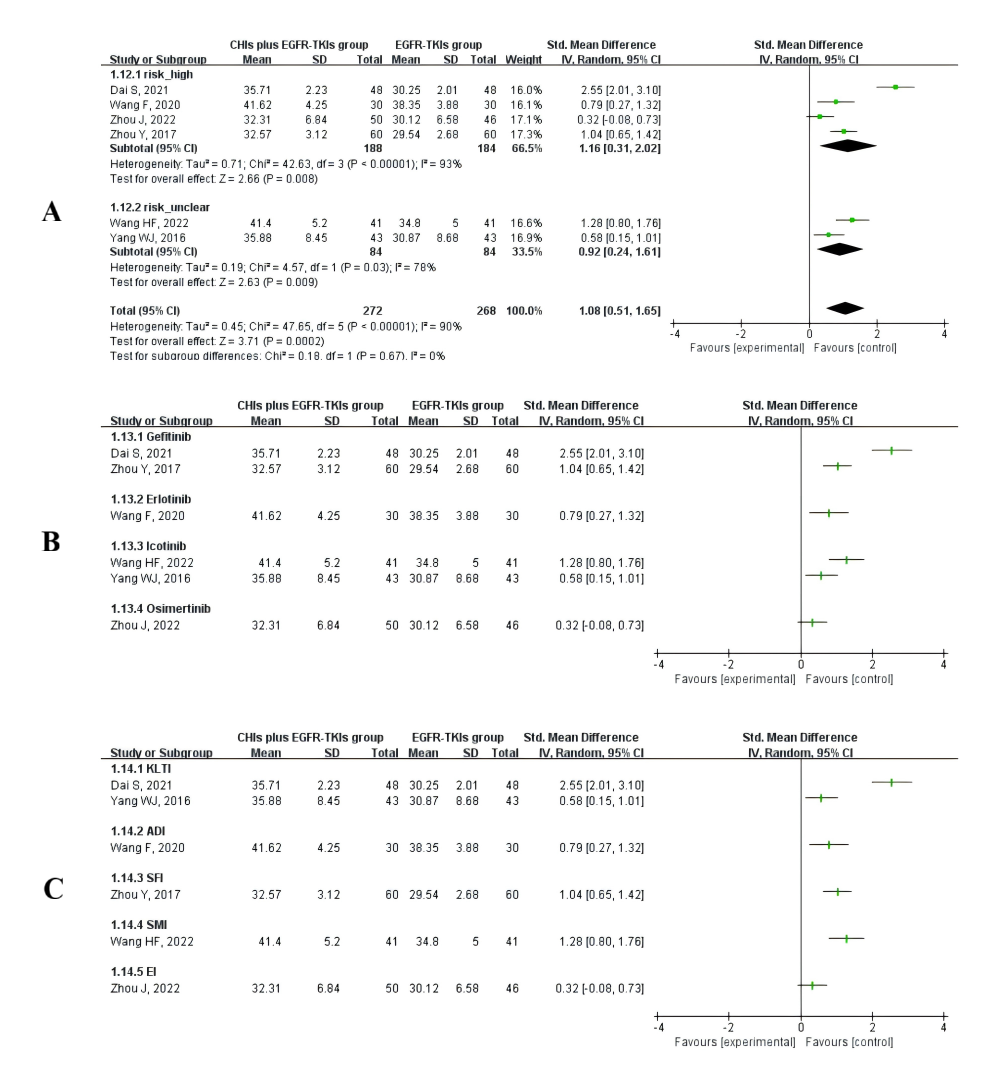


**Figure S2 A-C. Subgroup analysis of expression of CD4^+^ based on categorical variables.** A: methodological quality; B: EGFR-TKIs types; C: CHIs types.


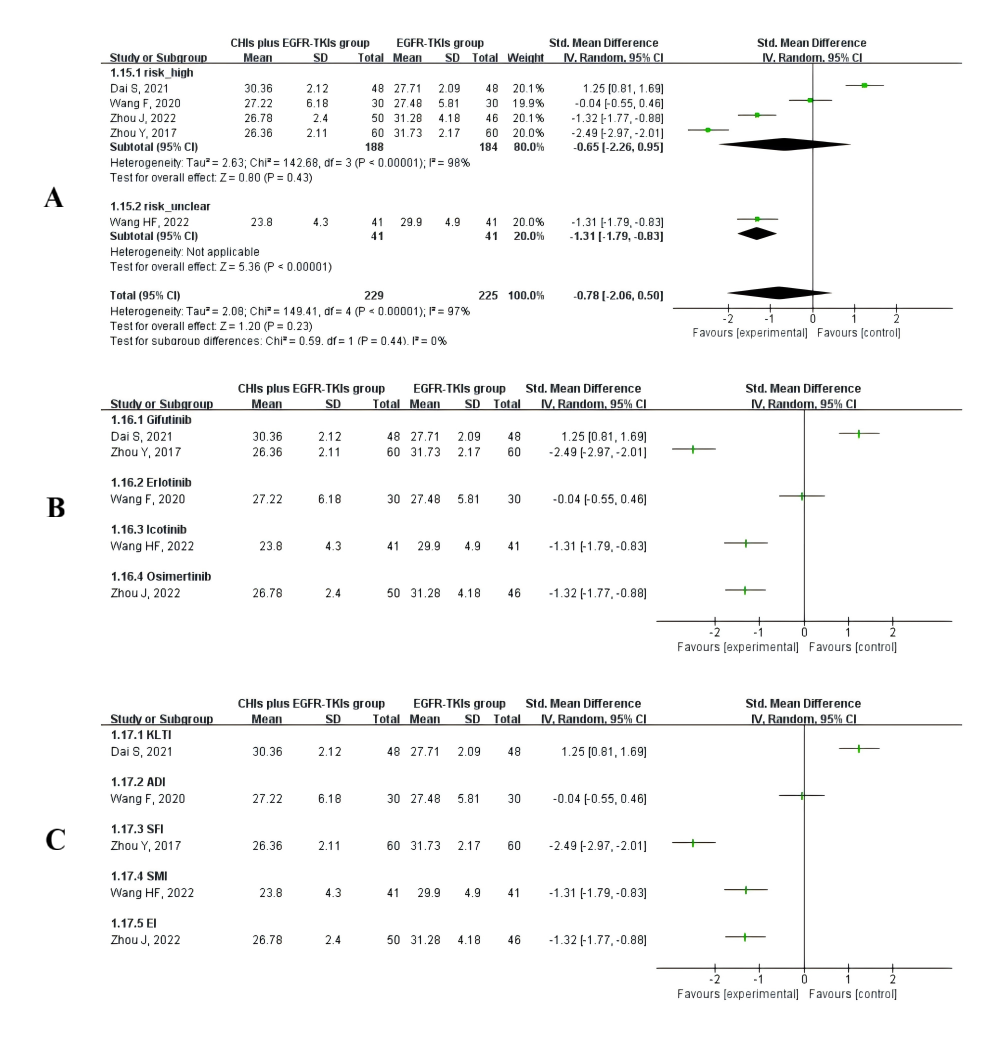


**Figure S3 A-C. Subgroup analysis of expression of CD8^+^ based on categorical variables.** A: methodological quality; B: EGFR-TKIs types; C: CHIs types.


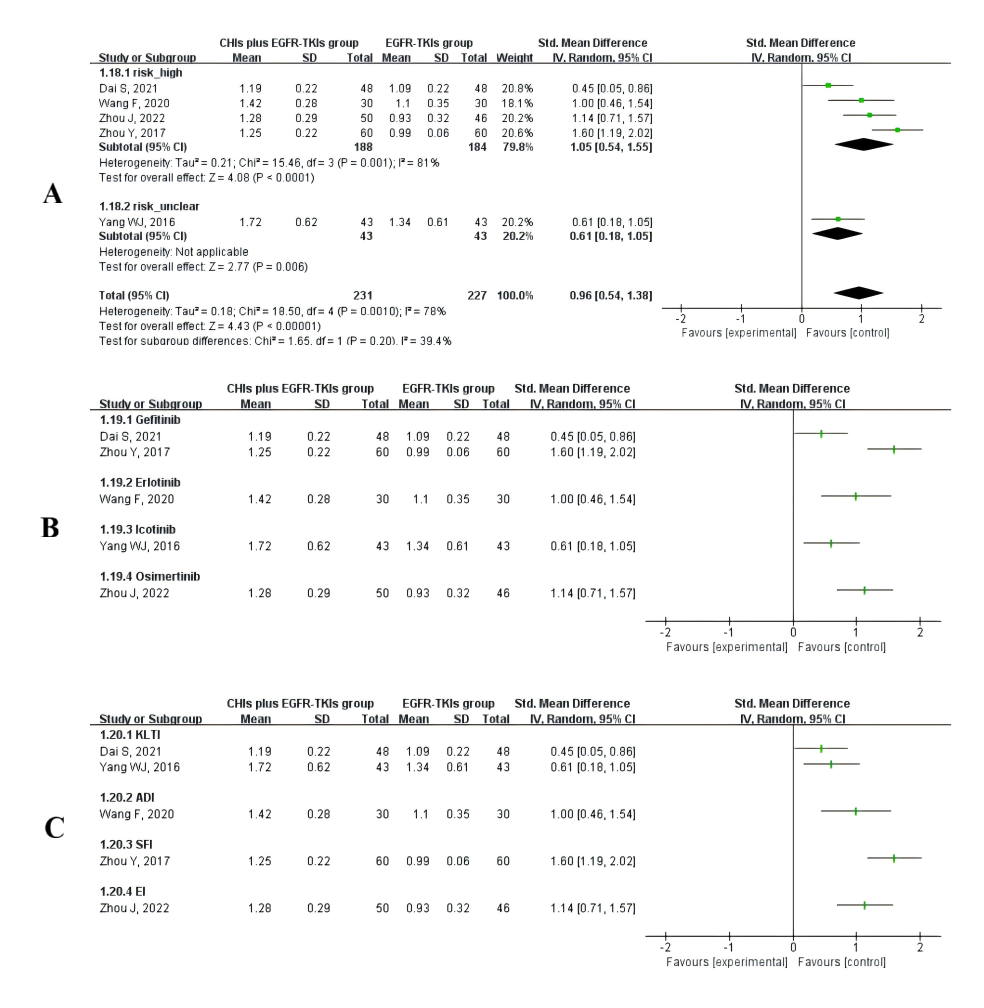


**Figure S4 A-C. Subgroup analysis of CD4^+^/CD8^+^ ratio based on categorical variables.** A: methodological quality; B: EGFR-TKIs types; C: CHIs types.

**
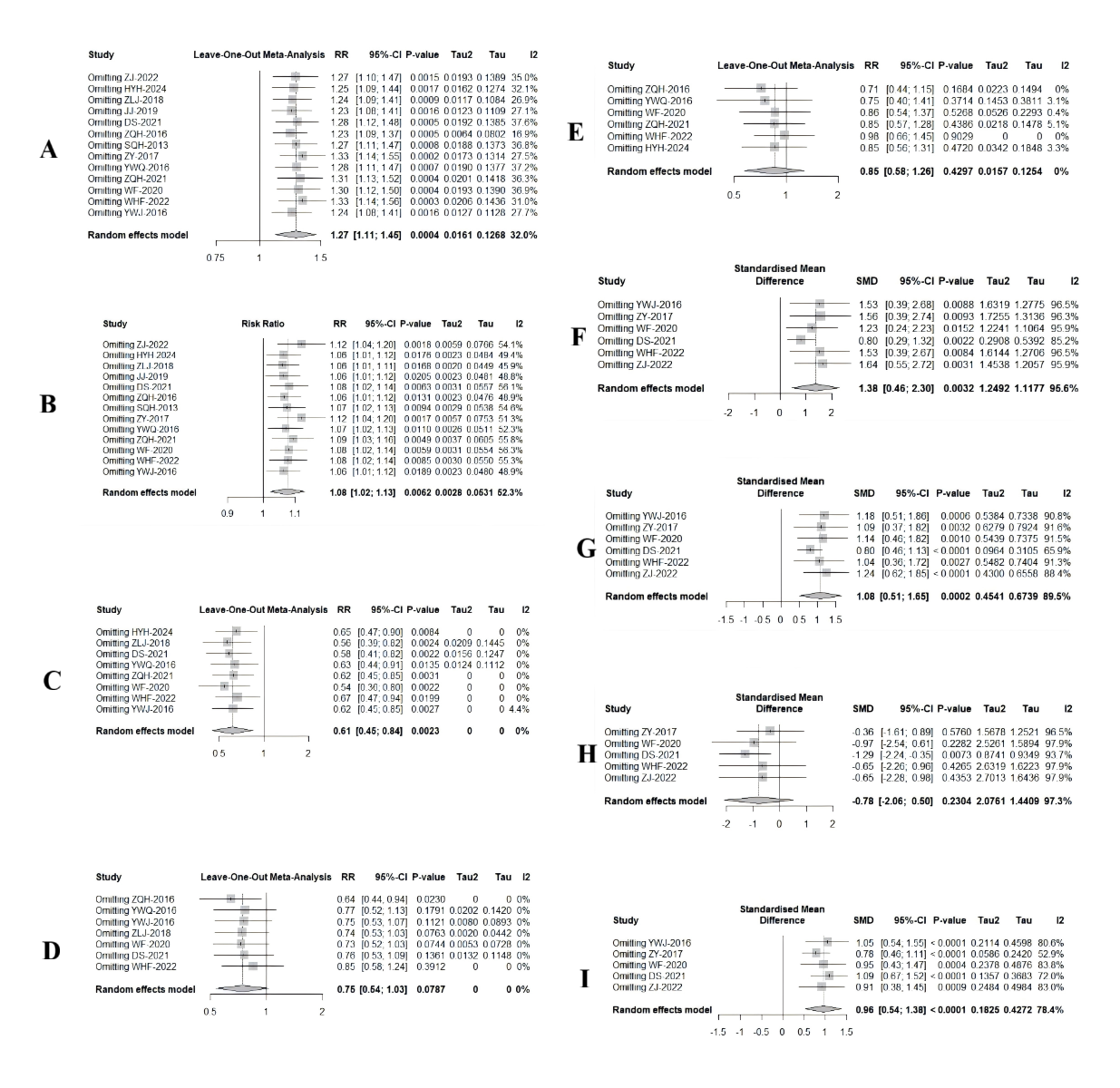
**

**Figure S5 A-I. Sensitivity analysis through exclusion of studies one-by-one.** A: ORR; B: DCR; C: dermatologic toxicities; D: gastrointestinal toxicities; E: hepatic insufficiency; F: expression of CD3^+^; G: expression of CD4^+^; H: expression of CD8^+^; I: CD4^+^/CD8^+^ ratio.
